# Refined annotation and assembly of the *Tetrahymena thermophila *genome sequence through EST analysis, comparative genomic hybridization, and targeted gap closure

**DOI:** 10.1186/1471-2164-9-562

**Published:** 2008-11-26

**Authors:** Robert S Coyne, Mathangi Thiagarajan, Kristie M Jones, Jennifer R Wortman, Luke J Tallon, Brian J Haas, Donna M Cassidy-Hanley, Emily A Wiley, Joshua J Smith, Kathleen Collins, Suzanne R Lee, Mary T Couvillion, Yifan Liu, Jyoti Garg, Ronald E Pearlman, Eileen P Hamilton, Eduardo Orias, Jonathan A Eisen, Barbara A Methé

**Affiliations:** 1 J. Craig Venter Institute (formerly The Institute for Genomic Research), 9704 Medical Center Dr., Rockville, MD, USA; 2 Institute for Genome Sciences, University of Maryland School of Medicine, Baltimore, MD, USA; 3 The Broad Institute, 7 Cambridge Center, Cambridge MA, USA; 4 Dept. of Microbiology and Immunology, Cornell University Veterinary Medical Center, Ithaca, NY, USA; 5 Joint Sciences Dept., Claremont Colleges, Claremont, CA, USA; 6 Dept. of Biomedical Sciences, Missouri State Univ., Springfield MO, USA; 7 Molecular and Cell Biology Dept., University of California Berkeley, Berkeley CA, USA; 8 Pathology Dept., University of Michigan Medical School, Ann Arbor MI, USA; 9 Dept. of Biology, York University, Toronto ON, Canada; 10 Dept. of Molecular, Cellular, and Developmental Biology, University of California Santa Barbara, Santa Barbara CA, USA; 11 UC Davis Genome Center, Section of Evolution and Ecology, University of California, Davis, CA, USA

## Abstract

**Background:**

*Tetrahymena thermophila*, a widely studied model for cellular and molecular biology, is a binucleated single-celled organism with a germline micronucleus (MIC) and somatic macronucleus (MAC). The recent draft MAC genome assembly revealed low sequence repetitiveness, a result of the epigenetic removal of invasive DNA elements found only in the MIC genome. Such low repetitiveness makes complete closure of the MAC genome a feasible goal, which to achieve would require standard closure methods as well as removal of minor MIC contamination of the MAC genome assembly. Highly accurate preliminary annotation of *Tetrahymena*'s coding potential was hindered by the lack of both comparative genomic sequence information from close relatives and significant amounts of cDNA evidence, thus limiting the value of the genomic information and also leaving unanswered certain questions, such as the frequency of alternative splicing.

**Results:**

We addressed the problem of MIC contamination using comparative genomic hybridization with purified MIC and MAC DNA probes against a whole genome oligonucleotide microarray, allowing the identification of 763 genome scaffolds likely to contain MIC-limited DNA sequences. We also employed standard genome closure methods to essentially finish over 60% of the MAC genome. For the improvement of annotation, we have sequenced and analyzed over 60,000 verified EST reads from a variety of cellular growth and development conditions. Using this EST evidence, a combination of automated and manual reannotation efforts led to updates that affect 16% of the current protein-coding gene models. By comparing EST abundance, many genes showing apparent differential expression between these conditions were identified. Rare instances of alternative splicing and uses of the non-standard amino acid selenocysteine were also identified.

**Conclusion:**

We report here significant progress in genome closure and reannotation of *Tetrahymena thermophila*. Our experience to date suggests that complete closure of the MAC genome is attainable. Using the new EST evidence, automated and manual curation has resulted in substantial improvements to the over 24,000 gene models, which will be valuable to researchers studying this model organism as well as for comparative genomics purposes.

## Background

*Tetrahymena thermophila *is a well studied model organism for molecular and cellular biology. Telomerase, self-splicing RNA, and the function of histone acetylation are some of the major discoveries made with this unicellular ciliated protozoan (reviewed in [1,2]). It was also the first member of the phylum Ciliophora to have its complete somatic (macronuclear, or MAC) genome sequenced [3]. Like other ciliates, *T. thermophila*'s MAC genome is a highly processed version of the germline (micronuclear, or MIC) genome, which is transcriptionally silent and responsible for direct transmission of genetic material to future sexual generations [4]. The transcriptionally active, amplified MAC genome consists of an estimated 180–250 chromosomes ranging from 20 kb to over 2 Mb long, collectively about 104 Mb. Purified MAC genomic DNA (strain SB210) was sequenced by the whole genome shotgun method to 9X coverage and assembled into 2,955 contigs and 1971 scaffolds that appear to represent a highly accurate and complete draft genome sequence [3].

Here we report significant progress toward genome finishing. Since the initial shotgun assembly, finishing efforts have succeeded in closing numerous sequencing and physical gaps. In addition, MIC/MAC comparative genomic hybridization (CGH) has identified 763 small scaffolds as probable MIC DNA contaminants. Together, these results reduce the number of MAC contigs and scaffolds to 1,826 and 1,177, respectively, and provide a greatly improved sequence assembly and foundation for structural gene annotation. Our closure efforts also confirm the low repetitiveness of the MAC genome and the absence of sequences highly related to invasive DNA elements [3]. These features make complete closure of this assembly feasible.

We also report here on improvements in *T. thermophila *genome annotation, which has presented certain challenges. First, comparative genomic data are extremely limited; although the MAC genome sequence and preliminary annotation of another ciliate, *Paramecium tetraurelia*, have also been released [5], these two organisms are only distantly related [6,7] (comparable to the mammal/arthropod split). In addition, *Tetrahymena*, like many ciliates [8], uses an alternative genetic code, in which UGA is the only stop codon and UAA and UAG encode glutamine [9], resulting in longer potential open reading frames in genomic sequence. Preliminary gene finding algorithms were trained using a small collection of *T. thermophila *cDNA sequences, supplemented with data from the genome sequence of the most closely related organism available at that time, the malaria parasite *Plasmodium falciparum *[10]. This *ab initio *gene prediction resulted in 27,424 putative protein-coding genes [3], over four times more than the most commonly studied unicellular eukaryotic model organism, *Saccharomyces cerevisiae *, and even more than many metazoans [11-13]. This high gene estimate is consistent with analyses of *T. thermophila *mRNA complexity [14] and with the even higher gene number prediction from *P. tetraurelia *[5]. We have sought additional direct evidence to refine the gene number estimate and gene structure predictions.

A powerful tool for structural gene annotation and expression profiling is the analysis of expressed sequence tags (ESTs), single-pass sequencing reads of cDNA clones [15,16]. Over 50 million ESTs have been deposited into the National Center for Biotechnology Information (NCBI) dbEST public database . Mapping of ESTs to an existing genome sequence by spliced alignment has allowed substantial refinement of gene models in a number of eukaryotic organisms (e.g. [17,18]). Besides providing a measure of direct confirmation of gene structure for those genes "hit" by an EST, the additional sequences of confirmed introns, and in some cases polyA-addition sites, provide better training data for gene finding algorithms to locate genes with low or specialized expression patterns that are not found as abundantly in the cDNA libraries used. Extensive EST sequencing is also effective in identifying alternatively spliced mRNA isoforms, the prediction of which by *ab initio *methods is in its early development [19,20] and has not been reported in protozoans.

Preliminary analyses of existing *T. thermophila *ESTs indicated that the *ab initio *predicted gene identifications are relatively robust [3]. However, a significant fraction of gene models appears to be flawed by, for example, incorrect splice site placement, missing exons, or improper merging or splitting of adjacent coding regions. Also, the cDNA libraries sampled represented only two physiological states, log phase growth in rich medium and sexual conjugation. Therefore, to improve structural gene annotation as well as to investigate gene expression under a wider variety of environmental and developmental conditions, we undertook a more extensive *T. thermophila *EST project.

cDNA libraries were constructed from cells treated under six different conditions that reflect key life cycle events as well as common interests of the *Tetrahymena *research community (see Table 1), including several growth conditions, starvation, and conjugation. Together with those previously reported [3], we have now generated 60,007 EST reads with valid alignments to the non-rDNA MAC genome assembly and examined them for patterns of condition-specific gene expression. Analysis of these ESTs provides direct evidence that over 40% of the *ab initio *predicted genes are indeed transcribed into mRNA and has allowed extensive refinement of the gene models, as described below. Alternative splicing appears to be rare in *Tetrahymena*, as also reported for *Paramecium *[21]. These refinements will significantly enhance the efficacy of transcriptome analysis and whole genome data mining. The revised protein-coding gene number estimate is 24,725.

**Table 1:** Characteristics of cDNA libraries and EST data.

**Cell treatment**	**Abbreviation**	**Vector(s)**	**Library prefix(es)**	**Total valid EST reads**	**5' reads**	**3' reads**	**No. of predicted genes hit**
Vegetative growth, rich medium	RCH	pcDNA3.1	TTB, TTC, TTD	10229	5270	4959	661

Vegetative growth, rich medium, plus Cu/Cd	HVM	pBluescriptIISK+	TTS	3160	3160	0	712

Vegetative growth, rich medium, plus TSA	TSA	pDNR-LIB	FTS	2651	2651	0	1810

Vegetative growth, minimal medium	MIN	pDNR-LIB	FMM	1809	1809	0	1157

Starvation	STV	pBluescriptIISK+	TT1	23321	12820	10501	4647

Conjugation	CNJ	pBluescriptIISK+, pDNR-LIB	TTE, FCO	18837	17813	1024	6568

**Total**				60007	43523	16484	9709

## Methods

### Comparative genomic hybridization microarray design

A *T. thermophila *microarray design (NimbleGen design id: 5314; design file: 2006-12-15_TetOrias_WG_CGH) was developed in collaboration with NimbleGen to, as evenly as possible, cover the TIGR November 2003 MAC genome sequence assembly . The array consists of 384,431 uniformly spaced approximately 60-mer isothermal probes. Mean probe start-to-start distance spacing is 257 bp; median is 260 bp. To avoid excessively repeated sequence or long homopolymer blocks (mainly A or T), individual probe spacing varies between 20 and 10,173 bp. Probes were designed for 1965 of the 1971 scaffolds. Three scaffolds (CH671888, CH671067 and CH671134) were excluded because they represent the rDNA MAC chromosome, and three small scaffolds (CH671770, CH671799 and CH671859, each < 2 kb) did not allow probe design because of high sequence repetition. Altogether the probes cover approximately one quarter of the entire assembled MAC genome sequence. To simplify the graphic display, NimbleGen reported the genomic probe positions and values for the 160 individual scaffolds larger than 195 kb, encompassing 80.927 Mb. The remaining 1908 scaffolds were reported as a concatenate; individual scaffold names and corresponding probe positions and scanned values were obtained using a list of scaffold starts supplied with the microarray design.

### Genomic DNA preparation for CGH

Cells obtained from four independent thawings of frozen *T. thermophila *strain SB210, the sequenced strain [3], were used as sources of purified MIC and MAC DNA. Cultures were independently grown as described [3]. MAC DNA was purified from three thaws and MIC DNA was purified from two thaws as described [22]. Southern blots of EcoRI-digested DNA preparations were probed with a region of the histone H1 gene containing MAC-destined and MIC-limited sequences [23] to verify minimal MIC and MAC DNA cross-contamination.

### DNA labeling, two-color hybridization, scanning, and data normalization

One of the purified MAC DNA preparation was chosen as the reference DNA for all the microarray hybridizations. The other two MAC preps and the two MIC preps were the sample DNAs. One of the MIC preps was used for two separate hybridizations, labelled MIC_116025 and MIC_114195 in Additional File 1: Normalized MIC and MAC array hybridization ratios. DNA labeling and hybridizations were performed as previously described [24]. For fluorescence intensity measurements, TIFF images were extracted using NimbleScan 2 software, NimbleGen System's image quantification and data analysis package. Grids were placed on each image and the quantification area for each feature was adjusted for optimal placement. After combining the signal intensity information with the genomic coordinate information, the Cy3 and Cy5 signal intensities were normalized to one another using "qspline normalization" [25].

### Microarray data analysis

For each microarray, we started with the NimbleGen-supplied file of normalized log_2 _ratios of sample:reference fluorescence measurements at every probe location. Normalized ratios were used to make more meaningful comparisons between different microarrays. For the two MAC-hybridized microarrays, the two scaffold ratios were simply averaged for each scaffold. To average the three MIC-hybridized microarrays, we did not take their simple average, as this would have given undue weight to the DNA prep used in two (duplicate) microarrays (MIC_116025 and MIC_114195; see above). Instead, for every scaffold, the scaffold-wide ratios in the duplicate microarrays were first averaged; the resulting value was then averaged with the value for the microarray hybridized with the second MIC prep DNA.

Transposon families in *T. thermophila *have been experimentally shown to be MIC-limited [26-28]. Scaffolds containing such known MIC limited sequences were identified using *T. thermophila *transposon genes available at GenBank. These are repetitive sequences and thus more likely present as contaminants of the MAC genome sequence assemblies. The nucleotide sequence of the *T. thermophila *MAC genome was searched by tblastn using the inferred peptide sequence of these genes as the queries and an Expect value (E value) limit of 0.001. For every scaffold, the hit with the lowest E value was used for further analysis.

### Gap closure

Most gaps and low coverage areas were sequenced by "primer walking" using custom primers on small to medium insert (1800–6500 bp) clones from the shotgun libraries [3]. Remaining areas were finished with custom primers on PCR products amplified from shotgun clones or genomic DNA. Repeat areas were resolved by producing transposon-mediated mini-libraries (New England Biolabs GPS-1 Genome Priming System; [29]) on shotgun clones spanning each repeat. In areas where the repeat was too large to be spanned by a single clone, a tiling path of clones was selected and each was transposon-tagged and assembled separately. All finishing assembly was done using the TIGR Assembler . To satisfy closure criteria, each base of finished sequence must be spanned by two clones or PCR products, and each base must have two underlying reads of high quality sequence. Areas where these criteria are not met are noted as exceptions.

### cDNA library construction and EST sequencing

A summary of cDNA libraries and EST sequencing is presented in Table 1. Libraries TTB, TTC, TTD (vegetative growth, rich medium), and TTE (conjugation) were constructed and sequenced as previously described [3]. All other libraries were constructed by Amplicon Express (Pullman, WA) from either cells preserved in RNAlater (Ambion, Inc.) or total RNA prepared using TRIzol. All cell incubations were at 30°C. For the FCO library (conjugation), equal aliquots of cells were harvested from a mating between strains CU427 and CU428 (two standard *T. thermophila *strains of the same inbred background as SB210) at 3, 6, 9, and 12 h after mixing. For all other conditions, strain SB210 was used. For the starvation library (TT1), RNA was prepared from cells harvested after 1, 2, 4.5, and 6 h in 10 mM Tris-HCl, pH 7.4 starvation buffer. For the heavy metal library (TTS), cells were grown to mid-log phase in Neff medium [30], and separate equal aliquots were treated with 11 μM CdCl_2 _or 500 μM CuSO_4 _for 1 h. For the minimal medium library (FMM), cells were grown to mid-log phase in minimal defined medium [31]. For the TSA library (FTS), cells were grown for 10 h (approximately 3.2 doublings, to mid-log phase) in rich medium plus 800 nM Trichostatin A. At this concentration, histone deacetylase activity is significantly inhibited (as evidenced by the appearance of acetylated histones in the normally transcriptionally silent micronucleus), but doubling time is only increased about 20% over control mock-treated cultures (unpublished data).

TT1 and TTS cDNAs were cloned directionally into the EcoRI (5') and XhoI (3') sites of pBluescriptIISK+ (Stratagene) and sequenced using T3 (5') and T7 (3') primers. Libraries FCO, FMM, and FTS were constructed using SMART technology and cloned into pDNR-LIB (Clontech) using SfiIA (recognition sequence: 5'GGCCATTACGGCC3') for the 5' end, and SfiIB (recognition sequence: 5'GGCCGCCTCGGCC3') for the 3' end. M13 forward primer was used for 5' sequencing. All reads containing the letters TF, TG, or TH in the suffix of their read IDs are from the 5' direction. All those containing TO, TV, T1V, or T7 are from the 3' direction.

### Reverse Transcriptase-PCR

Total RNA was isolated using TRIzol reagent (Invitrogen) from vegetatively growing *T. thermophila *strain SB210 and treated with DNaseI (Promega) for 30 min at 37°C. cDNA was synthesized from 2 ug of total RNA using SuperScriptII reverse transcriptase (Invitrogen) and reverse primers. PCR was performed using 1/10 of resulting cDNA with forward and reverse primers flanking the splice sites in question and Taq polymerase, with 28 cycles of 94°C for 45 sec, 56°C for 45 sec, and 72°C for 30 sec.

### EST alignment and assembly

Sequences were trimmed for low quality regions, vector contamination, and poly-A tails using SeqClean  and the UniVec database . In addition, contaminating rRNA sequences were removed from the data set by screening against the published rDNA sequence [3,32] using the Program to Assemble Spliced Alignments (PASA) [17]. The remaining EST sequences entered the PASA pipeline and were aligned against the most complete genome sequence (see Genome Closure below) using BLAT, sim4 and GeneSeqer, as previously described [17]. Alignments were validated by strict criteria, requiring GT/AG consensus donor/acceptor splice junctions and at least 95% sequence identity over at least 90% of the sequence length. ESTs aligning to the genome with putative introns less than 20 bp or greater than 10,000 bp in length were excluded (this only affected 18 ESTs).

### Gene model refinement

Since the preliminary gene annotation [3], an interim release of gene models was made available on the *Tetrahymena thermophila *Genome Project ftp site  on 8/31/2006. Both that reannotation and the currently reported gene model refinement made extensive use of EVidenceModeler (EVM), an automated tool that reports eukaryotic gene structures as a consensus of all available evidence, weighted according to the form of evidence [33]. In *Tetrahymena*, EST alignment by PASA was given the highest weight, followed by similarity of predicted peptide sequences to sequences of other organisms, then *ab initio *gene predictions. Gene Ontology (GO) terms were attributed to gene products with Pfam domains having scores above the specific trusted cutoff .

## Results and discussion

### Determining the nuclear source (MIC vs. MAC) of every scaffold

Our shotgun sequencing libraries [3] were constructed from DNA of macronuclei purified by standard differential centrifugation methods [34] that do not wholly eliminate MIC contamination. We are aware that minor MIC DNA contamination of the whole genome assembly occurred, but the exact extent of contamination was unknown. Building a clean database of only MAC scaffolds would greatly facilitate physical mapping [35] and genome finishing, and would also be useful for annotation and for understanding how the MAC genome is formed from its MIC precursor. The MIC genome is estimated to be approximately 15% larger than that of the MAC, much of the extra DNA being repetitive [36]. MIC-limited DNA is found dispersed throughout the MIC genome at as many as 6,000 internally eliminated sequence (IES) sites [37]. These IESs are removed through an epigenetically controlled whole genome rearrangement process that occurs during the sexual conjugation pathway [38]. Our preliminary analysis [3] supported the idea that this IES removal acts as a genome defense against invasive DNA elements and not as a barrier against repetitive DNA *per se*, as in Repeat-Induced Point Mutation in *Neurospora *[39]. The results presented below provide further support that sequences highly related to invasive transposons are found exclusively, or nearly so, in the MIC.

Because of limited sequence coverage of the MIC contaminating DNA, it was assembled into only small scaffolds. In the initial November 2003 whole genome assembly, scaffolds smaller than 3.3 kb account for two thirds (1335/1971) of all scaffolds, but only 1.8% of the assembled sequence. Analyses of transposon-related sequences, including known MIC-limited *T. thermophila *transposons [26,27], showed that the great majority match to very small scaffolds [3]. HAPPY mapping ([40]; a physical linkage method currently being used to join *Tetrahymena *scaffolds into complete MAC chromosomes) provides independent evidence that small scaffolds are enriched for MIC-limited DNA (Orias & Hamilton, unpublished observations alluded to in [35]). All underrepresented markers in the HAPPY mapping panel (present at levels significantly lower than expected for single-copy MAC DNA sequences and therefore most likely representing the diploid MIC rather than the polyploid MAC) were located on scaffolds < 3.3 kb in length. Indeed, at least 40% of those scaffolds match known *T. thermophila *MIC-limited sequences [26-28].

To identify MIC DNA-containing scaffolds, we used array-based Comparative Genomic Hybridization (CGH) to measure MIC-specific to MAC-reference hybridization intensities of nearly all scaffolds. We designed a microarray with more than 380,000 unique probes that together cover about 25% of the entire genome assembly (see Methods for details). To this microarray we hybridized three purified MIC and two purified MAC DNA samples (labeled with different fluorescent dyes) isolated from independent cultures of strain SB210. A third MAC DNA preparation was used as the reference DNA in each of these two-color hybridizations.

Purification of MIC or MAC DNA is based on physical separation of nuclei and is not complete, although, because of higher MAC ploidy, MAC DNA preparations are purer. Still, purified MIC DNA is greatly enriched for MIC-limited DNA sequences relative to purified MAC DNA (MAC-retained sequences are present in both). The fluorescence intensities were expressed as the normalized log_2 _ratios of sample:reference DNA hybridizations. A ratio was determined for each scaffold by averaging the log_2 _ratios obtained at every probe position on a given scaffold (between 1 and 8,303 probes, depending on the length of the scaffold). MIC ratios were separately obtained for each scaffold for each of the three MIC DNA-hybridized arrays and averaged, as described in Methods; the same was done for the two MAC DNA-hybridized arrays. The scaffold ratio for each individual hybridization and the average MIC and MAC ratios are included in Additional file 1: Normalized MIC and MAC array hybridization ratios.

**Figure 1:**
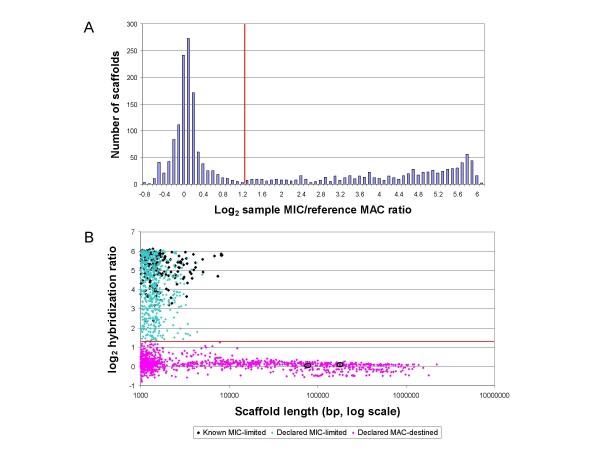
**Results of MIC/MAC comparative genomic hybridization**. A: Distribution of MIC scaffold ratios. Red line: proposed separation of MAC-destined (maD) DNA scaffolds (on the left) and MIC-limited (miL) scaffolds (on the right). B: Scatter plot of MIC scaffold ratios as a function of scaffold length. Pink and aqua points: maD and miL DNA, respectively, by the log_2 _ratio criterion in Figure 1A. Black diamonds and small black circles, respectively: miL and maD scaffolds with high sequence identity to miL transposon genes. The maD distribution is more diffuse as the length decreases to the minimum scaffold length (1,000 bp). This is attributed to the fact that the number of probes is roughly proportional to scaffold length. Given a uniform intrinsic variability in hybridization ratios for each probe, the variance of the scaffold means is expected to vary inversely with scaffold length. The secondary peak in the maD distribution (around log_2 _ratio = -0.45) in 1A and the multimodality of the maD distribution in 1B (most clearly seen for scaffolds > 50 kb) are caused by the partial loss of MIC chromosome segments in the cells used for the MIC DNA preps (Orias and Hamilton, unpublished observations).

The numerical distribution of scaffolds by MIC ratio is shown in Figure 1A. Most scaffolds are found in a large peak at a log_2 _ratio near 0 (i.e., 1:1 hybridization ratio of purified MIC and MAC DNA), as expected for MAC-destined DNA (since all MAC sequences are derived from, and hence also present in, the MIC). To further validate that this peak represents MAC-destined scaffolds, MIC ratios were plotted as a function of scaffold length (Figure 1B). The largest scaffolds showed a uniform depth of coverage in the shotgun sequencing project (Fig. 4 in [3]) and are thus clearly composed of MAC-destined DNA. As expected, the largest scaffold data points hover near the 1:1 hybridization ratio (log_2 _= 0).

A red line has been drawn in Figure 1A at a log_2 _ratio of 1.3, the location where the tail of the MAC-destined DNA peak ceases to decrease. To our best current approximation, a log_2 _ratio higher than 1.3 indicates the corresponding scaffold is MIC-limited. Ratios higher than 1.3 are diffusely distributed, varying up to a log_2 _MIC: MAC hybridization intensity ratio of 6.2 (i.e. > 70:1). Factors that may account for this diffuseness include the documented copy number variation of MIC-specific elements [41], cross-hybridization between MIC-specific elements and repetitive MAC-retained sequences (Hamilton and Orias; unpublished observations), and intrinsic probe hybridization variability exacerbated by the small size of these scaffolds and thus the small number of array probes in each of them.

To further support our conclusions based on comparative hybridization, we identified scaffolds that match known MIC-limited *T. thermophila *transposon genes (see Methods and Additional File 1). 224 scaffolds that matched such genes with E values less than 1.0E-25 were found. All but two are small scaffolds (< 10 kb) and fall above the 1.3 value for the averaged MIC:MAC log_2 _ratio (black diamonds, Fig. 1B), providing strong validation of the array approach to identify MIC limited sequence. The two exceptions (CH670409, 78 kb and CH445591, 180 kb; black circles, Fig. 1B) fall well below the E value upper boundary (1.2E-306 and 7.5E-76, respectively). Since the stringency of the match is so high but the scaffolds are clearly in general MAC-destined, we suspect that these two exceptions represent rare cases where the Celera assembler co-assembled MAC-destined and contaminating, high copy number MIC-limited sequence (but for an alternative explanation, see below). We have seen another rare example of MIC:MAC DNA co-assembly: Chromosome breakage sequence Cbs4R-7, which is MIC-limited, was assembled at one end of scaffold CH670376 (Hamilton & Orias, unpublished observations).

As the stringency of transposon matches decrease (i.e., E value > 1.0E-25), there is a dramatic increase in the number of matches to large scaffolds that, by the CGH criterion, are clearly MAC-destined (see Additional File 1). Co-assembly of MAC-destined and MIC-limited DNA remains a possible explanation for some of these hits. Alternatively, some transposon copies, or segments thereof, may have become permanently MAC-destined. This may happen with evolutionary time, as their sequences degrade to the point that they can no longer be recognized by the scnRNAs that guide IES removal [42], even if transposon sequence-relatedness is still detectable by blast search. As an intermediate stage in this trajectory, such segments may still be generally MIC-limited but failed to be removed during the differentiation of the MAC in the particular cell line that was sequenced. Better understanding of these possibilities can be gained once the MIC genome sequence becomes available and by comparison of multiple independently derived MAC genomes.

To summarize, the vast majority of MIC-limited scaffolds are in all likelihood correctly identified. For most of the scaffolds, the identification remains statistical in nature and there is some possibility that a few scaffolds near the threshold have been misclassified. Together, the 763 MIC-limited scaffolds identified here represent about 1.2% of the prior assembled genome sequence and about 8% of the total estimated MIC-limited sequence (about 15 Mb [36]). After removal of the 763 MIC-limited scaffolds and other closure efforts (see below), the complete span of *T. thermophila *scaffolds is 103,085,054 bp, a decrease of 1,109,369 bp from the previously published figure [3].

### Targeted gap closure

Multiple, independent lines of evidence indicate that the published whole genome assembly is of high quality and completeness [3,35], but, as with all draft genome sequences, many gaps remain. Because these gaps contain or interrupt an unknown number of genes, their closure helps to obtain as complete a gene annotation as possible. Additional benefits of closure include placement of genes in a more extended chromosomal context and the correlation of physical and genetic maps, both for the MAC and ultimately the complete MIC genome. Closure of the *Tetrahymena *MAC genome is somewhat complicated by the presence of as many as 200 or more chromosomes, but made simpler by the low levels of repetitive sequence.

Initial assembly of bulk (non-rDNA, non-mitochondrial DNA) MAC shotgun sequence reads using the Celera assembler [43] generated 1,971 scaffolds, composed of 2,955 contigs [3]. Following the CGH analysis reported above, the number of MAC-derived scaffolds can be reduced to 1,177. Of these, 125 scaffolds are capped with telomeric repeats at both ends and thus represent complete MAC chromosomes. An additional 120 scaffolds are capped with telomeres at one end. Remaining gaps can be divided into two types: sequencing gaps and physical gaps. Sequencing gaps occur between the contigs of a scaffold and result from no, low, or poor quality sequence coverage of regions that are, nevertheless, contained in known clones from one of the genomic DNA sequencing libraries. Therefore they can generally be closed by methods such as primer walking. Physical gaps separate scaffolds and can occur stochastically or result from the absence of sequence from regions that are underrepresented because of incompatibility with *E. coli *cloning, or repetitive sequence regions that cannot be unambiguously resolved by the Celera assembler. By visual inspection and resolution of such terminal repetitive regions, a number of scaffolds were linked into an additional 14 complete chromosomes, as shown in Additional file 2 (Summary of genome closure progress), and the lengths of four single telomere-capped scaffolds were extended.

*Tetrahymena *closure efforts focused first on finishing to high standards of quality the largest scaffolds that are capped by telomeres at both ends, i.e. full chromosomes with no physical gaps. Additional file 2A lists 123 chromosomes that are now completely finished, comprising 54,470,278 bp of sequence, approximately 53% of the estimated full genome length. Because we did not preselect "trouble-free" sequencing gaps, our success with this unbiased approach supports the idea that highly repetitive regions will not present a serious challenge to complete closure of sequencing and perhaps physical gaps.

Substantial progress was made in closing an additional seven complete chromosomes, which are listed in Additional file 2C as "Complete Chromosomes, with Exceptions". The exceptions represent small regions that do not meet our quality control standards (see Methods). Six of these regions, ranging in length from 47 to 307 bp, are covered by only a single clone, sequence, or high quality sequence. Four other regions, estimated at between 161 and 651 bp, have no sequence coverage. These gaps are represented as a corresponding number of unknown bases (Ns) in the assembled sequences. In two cases, sequence up to the telomeric repeats has not been determined, although the close proximity of telomeres has been confirmed by PCR. These seven nearly finished chromosomes comprise approximately 4.8 Mb of additional sequence. In addition to the complete chromosomes, progress was made on closure of four large, single telomere-capped scaffolds. Additional files 2B and 2D list, respectively, three fully closed single-capped scaffolds and one "Single-Capped Scaffold, with Exception", which has a single sequencing gap of approximately 435 bp. Together, these comprise approximately 3.5 Mb.

Following closure, approximately 60% of the *Tetrahymena *MAC genome is now completely finished, or finished with minor exceptions in coverage or small gaps. Of an estimated 984 sequencing gaps in the initial Celera assembly, 327 have been closed, in addition to 26 physical gaps. Six of the linkages by which scaffolds were extended have been independently confirmed by published HAPPY mapping links [35], and others have been confirmed by unpublished HAPPY results (E.O., E.P.H. and P. Dear).

### EST sequencing and analysis

Previously reported EST sequencing efforts [3,44] were confined to two cell conditions – log phase growth in rich medium and conjugation. To sample a wider variety of ESTs, cDNA libraries were constructed and sequenced from these as well as several other conditions (see Table 1). *Tetrahymena *grows rapidly in simple rich medium (primarily proteose peptone), with a doubling time of approximately two to three hours [45], but can also be grown in simple chemically defined medium that includes a number of amino acids, nucleosides, salts and vitamins [31,46]. Log phase cells grown in both rich and minimal media were used for library construction, as well as log phase cells in rich medium plus heavy metals, which induce expression of metallothioneins and other stress proteins [47], or the histone deacetylase inhibitor Trichostatin A, which is known in other organisms to activate certain genes by causing hyperacetylation of chromatin [48,49].

In addition, two cDNA libraries were constructed from mRNA collected at various time points of conjugation and starvation. The sexual conjugation pathway of *Tetrahymena *has been extensively studied [7], in large part because it is during this period that the genome-wide DNA rearrangements that shape the macronuclear genome occur [37]. Conjugation is an inducible process that, under ideal conditions, can be highly synchronous in large populations [50]. A number of genes have been identified (several with essential roles in genome rearrangement) that are expressed solely or predominantly during conjugation (e.g. [51-54]). Cell cultures are prepared to initiate conjugation by starvation in very dilute buffer (typically 10 mM Tris), a treatment that also induces morphological alteration to a "fast swimmer" cell shape [55], changes in chromatin structure [56] and histone modification [57], and general and specific changes in mRNA abundance [14,58].

A total of 80,258 successful EST reads were obtained from the nine cDNA libraries listed in Table 1, representing the six different cell conditions described above. The sequences were trimmed for vector contamination and low quality regions as described in Methods. All ESTs were submitted to the NCBI dbEST public database (Accession numbers: DY676394 - DY684793, CN587913 - CN599370, CX571148 - CX592097, EC268787 - EC275251, EV826049 - EV849892, FF562771-FF578826), as well as to the Taxonomically Broad EST Database (TBestDB; ), an EST repository focused on protists. Over 11,000 sequences that align to the rDNA locus [32] were removed from the pool, and all remaining ESTs were aligned to the whole genome assembly using PASA [17]. After removing sequences that failed alignment and splice site validation criteria (see Methods), 62,275 ESTs that align to bulk (non-rDNA) MAC chromosomes remained. By visual inspection, we excluded an additional 2,268 ESTs from the set used for reannotation purposes because of inconsistencies (e.g. 5'-3' orientation, adjacent gene overlap) with other strongly supported gene model evidence (including other EST evidence), leaving 60,007 ESTs (see Table 1), which were used for all subsequent analyses. Sequences of the 60,007 validated ESTs are available for download at .

In addition to contamination by rRNA, ESTs that failed to align with the bulk MAC genome assembly could possibly originate from transcripts of DNA regions not contained in the assembly, namely sequences located in assembly gaps, MIC-limited DNA, and the two or more chromosomes known to be lost during early vegetative growth following conjugation [59]. It has been repeatedly reported [60-63] that, at least during vegetative growth, there is no detectable expression of the MIC genome. During conjugation, non-genic, bidirectional transcription of germline-limited DNA occurs [64], but there is no evidence such transcripts are poly-adenylated (D.L. Chalker; pers. comm.), and thus, they should be underrepresented in our polyA-selected cDNA libraries. None of our ESTs aligned to the 763 scaffolds identified, as described above, as MIC contaminants, which account for an estimated 8% of MIC-specific sequences. We also examined the ESTs that failed our strict alignment criteria. The vast majority of ESTs derived from genome regions not represented in our assembly would be expected to fail both alignment criteria of greater than 90% identity over greater than 95% total sequence length, but none of our EST reads did. Most alignment failures appear to be the result of chimeric inserts. Thus, we have not detected any evidence for transcription of germline-limited DNA, chromosomes lost during early vegetative growth, or unidentified genes found in unsequenced assembly gaps.

The principal purpose of this EST sequencing project was to gain experimental evidence on the structure of as many genes as possible; therefore we analyzed the ESTs in batches as they were generated to search for signs of redundancy in the libraries and adjust the proportion of reads obtained from each. Compatible overlapping EST sequences (i.e. with no splice junction disagreements) that met all alignment criteria were clustered using PASA into 12,814 clusters (see Additional file 3: EST to gene mapping). Overall, 49% of these clusters were singletons and only 9% were represented by greater than ten EST reads. These results indicate that the libraries were far from being saturated and that further sampling would generate additional data concerning gene expression. The PASA clusters mapped to a total of 9,709 predicted genes. In 7,168 cases, just one PASA cluster mapped to each predicted gene, but between two and eight PASA clusters mapped to the remaining 2,541 predicted genes to which any ESTs were mapped (see Additional file 3). Most of these cases are the result of nonoverlap between the clusters, which is not surprising, given the fragmentary nature of EST evidence and that many clones were sequenced from both the 5' and 3' directions. Some of these nonoverlap cases may represent incorrect gene predictions that should be split into two adjacent genes. However, in the absence of confirmatory evidence, e.g. full-length cDNA sequence or reliable comparative genomic data, we did not update gene annotations by splitting any gene models.

In contrast, PASA reported 466 cases of overlapping, but incompatible, sets of between two and five assemblies that represent potential examples of alternative splicing within protein-coding regions. Alternative splicing within predicted mRNA untranslated regions, which was previously reported in one *T. thermophila *gene [47] using the same set of EST evidence described here, was not examined in this study. There has been only one published report of alternative splicing in *Tetrahymena *affecting a predicted open reading frame [65]. Each of the 466 putative new examples was visually inspected. Most alternate assemblies consisted of only a single EST in which one or more introns was unremoved; inadvertent cDNA cloning of immature mRNAs is a potential explanation for these isolated examples. In a number of other cases, a single EST showed evidence for usage of an alternative splice junction, resulting in either in-frame insertion or deletion of between one and seven codons, alternative start codon usage, or frame shift. Although such outcomes might be programmed, the supporting evidence of only a single EST suggests they may instead represent rare splicing errors. That the coding potential outcomes were generally either minor (e.g. insertion of one or two codons) or drastic (long-range frame shift) tends to support this view, although additional evidence is necessary to reach a final conclusion.

Stronger evidence for alternative splicing was found in ten cases, summarized in Additional file 4: Putative cases of alternative splicing. In these cases, the alternate assembly consists of between two and eight ESTs from independent cDNA clones. Six of the ten cases represent alternate intron removal or retention; the other four represent alternate splice junction usage (two 5', two 3'). The consequences of such putative alternative splicing events on coding potential include insertion or deletion of between 9 and 28 codons, and limited-range N- or C-terminal frame shifts. To confirm these alternative splices, we performed RT-PCR on vegetative cell RNA. We successfully obtained amplification products for eight of the genes in question (see Additional file 4), and, in each case, the alternative splices were confirmed (data not shown). In addition, for the four cases of alternate splice junction usage, another amplification product corresponding to unspliced mRNA was detected.

We conclude from this analysis that alternative splicing is uncommon in *T. thermophila*, at least under the growth conditions we have examined, as has also been observed in *P. tetraurelia *[21]. Alternative splicing has been posited as a means of encoding more information in the genomes of complex metazoans, such as humans, that have only marginally higher protein-coding gene counts than *Tetrahymena*. Recent studies [66] suggest most alternatively spliced human transcripts may not encode functional protein products, and that these alternate transcripts may simply be tolerated but provide potential for rapid evolutionary changes. It remains to be seen whether the apparent rarity of alternative splicing in ciliates is directly related to their relatively high gene count and, if so, by what mechanism.

**Figure 2:**
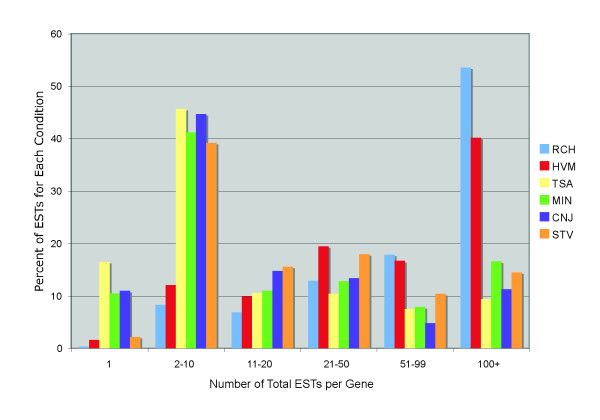
**Distribution of EST gene hits**. The x-axis is divided into bins by the total number of validated ESTs (from all libraries) hitting a given gene. The y-axis depicts the percent of ESTs from each of the six conditions that fall into the indicated x-axis bin. For example, the bin containing genes matched by between 2 and 10 ESTs contains 8,426 matches from the conjugation condition (TTE and FCO libraries). The total of all CNJ ESTs is 18,837 (see Table 1). The percent of total CNJ ESTs in this bin is therefore 8,426/18,837 = 44.7%. Abbreviations as in Table 1.

Figure 2 shows a measure of the redundancy of each library (or set of libraries), relative to the entire pool. Measured as a percentage of total valid EST reads from that condition, most of the conditions generated novel gene "hits" at comparable rates. However, the rich medium and heavy metal libraries displayed higher redundancy, with over 53% and 40%, respectively, of ESTs corresponding to genes that were hit by 100 or more total ESTs (from any condition). These two conditions also resulted in the lowest number of total predicted genes hit (Table 1, final column). The rich medium and heavy metal EST reads were particularly dominated by a few genes that each made up 1% or more of their totals; 19 and 18 such predicted genes accounted for over 48% and 33%, respectively, of all rich medium and heavy metal ESTs. The greatest number of reads with consistently low redundancy, as well as the highest number of gene hits, was obtained from the conjugation and starvation libraries. These differences may reflect as much or more on the relative quality of the cDNA library preparations as on true differences in mRNA complexity, which would be more reliably determined through expression microarray analysis.

### Improvements to structural annotation

Despite many advancements, automated gene finding in eukaryotic genomes remains a challenge [67]. In the case of *Tetrahymena*, the lack of substantial comparative sequence data from near relatives and/or EST data led us to the use of *ab initio *methods that, while sufficient for a general analysis of genome contents [3], do not provide gene models of satisfactory quality for a widely studied model organism. With the EST evidence here reported, many automated updates were made using the tools described below, but assessment of multiple forms of evidence still required manual intervention in many cases.

Initial gene finding was done using TIGRscan [68] and custom parameters to generate 27,424 protein-coding gene models [3]. For this study, gene finding was repeated using the programs Genezilla (; based on TIGRscan), GlimmerHMM [68], and Augustus [69,70]; Genezilla was the most sensitive of these and also performed with high accuracy, on the basis of accumulated EST evidence. As more EST sequences became available, as well as the genome sequence of *P. tetraurelia *[5], we used EVidenceModeler (EVM; ) [33] to generate consensus gene models from an automated weighted evaluation of the various independent pieces of structural evidence. EST evidence was by far the most valuable source of data to improve the gene models and increase sensitivity. Many models were short; as a conservative cutoff, we eliminated 1,577 models with a predicted coding length of less than 40 amino acids.

In addition to using automated methods, 2000 gene models were manually curated by proceeding from end to end of the largest scaffolds. An additional 725 gene models that lacked an ATG start codon were examined and corrected. Gene models for which EST sequence alignment was problematic (e.g. the 2,268 excluded ESTs described above) were visually inspected, resulting in some cases in gene model mergers and/or refinements. In some cases, updates to the gene models implemented by EVM, which relies heavily on PASA gene model predictions, caused spurious consequences. ESTs may be successfully mapped to a location on the genome assembly, but, in the absence of a polyA-tail to orient the direction of transcription, the PASA algorithm will simply look for the longest single exon ORF on either strand to make this determination. Usually, this longest ORF is on the correct strand and corresponds to the gene model predicted by gene finding algorithms, but occasionally it is on the opposite strand, and an erroneous gene model overlapping the original is automatically generated. Such cases were reviewed and manually corrected. We also incorporated published annotation corrections based on proteomic evidence [71,72].

Following these automated and manual structural curation steps, our latest estimate of protein-coding genes in the *T. thermophila *genome is 24,725. These gene models have been submitted to Genbank and are available on the project's ftp site  and from the *Tetrahymena *Genome Database (TGD; ; [73]). Overall features of the gene models are similar to those reported previously (Table 2 and Figure S3 in [3]). 71% of gene models contain at least one intron, with an average length of 152 nucleotides. The revised average number of introns per gene is 3.6.

**Table 2:** Reannotated *T. thermophila *selenoprotein homologs.

**Gene Type (Putative Selenoprotein)**	**Genbank ID**	**3' UTR Containing Putative SECIS (5'-3')**	**ESTs**
Glutathione Peroxidase	TTHERM_00141160	AUUUUCAAAUAUUGAAAACUAAAAUGUUAAAUGAAAGAUUAUUUUUAAAUUUGUAAAAAAGAAAUAAUUUUGAAAAAAAUAUUAUUUUAGUUAGU	20 STV, 1 CNJ, 1 HVM, 1 MIN

Glutathione Peroxidase	TTHERM_00279820	GAUAAAAGAGAUAUCAUUCAAUGAUAGCUUUAUAAUUAAAUCUUUAAUAGAAGUUAUAAGGUUUGAAGCUAAUGAGCUCUAUUAUC	2 STV, 2 CNJ, 1 TSA

Thioredoxin Reductase	TTHERM_00486500	AAUUUAUAUAUCUUAAAGAUGUAUAGUAUAAUGAUAGCAAAUCUCGAAAAUCUUAGGAUUGGGAUUAGGCUUGAAUAGUCAGAGUAAUAAGAGUAUUUAUUA	0

Thioredoxin Reductase	TTHERM_00823430	UCUAAUAUGGAAAAUGACGAAUUUAGUCUAAACUGUAAAACAGGGAUUAAAUUCUGAA	6 STV, 1 TSA

Thioredoxin Reductase	TTHERM_00047660	AUACCUUCAACUGGUAGGAAUAUAAUGAUUAGAGAACUCCUAACCUCACUGAGGAGGGUUUUCUAUGAGGCAAGAUUAUUGAUUUUGUUGUAG	164 RCH, 11 STV, 5 CNJ, 1 HVM, 1 TSA, 1 MIN

Selenophosphate Synthetase 2	TTHERM_00522580	AAGUAUCAUUUCUAAAAUGAUGCAAAUUAUUACCUGAAACUCUAAAAGAGAAGGAAUUUGCUGAGAAAAAAAAUGAAAAUGAUAACUU	7 STV, 3 CNJ, 1 HVM, 1 TSA

**Other Reannotated Genes**			

Glutathione Peroxidase	TTHERM_01099010	N.A.	0

Thioredoxin Reductase	TTHERM_00723630	N.A.	5 STV, 2 TSA

SelO	TTHERM_00852990	N.A.	4 STV

The first six are putative selenoproteins. EST library condition abbreviations are as in Table 1

Because of the numerous gene model updates, both manual and automated, performed over an extended time period, including 5' and 3' extensions, splicing changes, frame shifts, and mergers, it is difficult to fully characterize the nature and degree to which the gene models have been altered as a whole from the first published set [3] to the present. Nevertheless, to make an estimate, we performed a BlastP search of each of the updated predicted peptide sequences (query) against the preliminary set (target) to find the highest scoring match (an "all vs. all" search). The full results are presented in Additional file 5: BlastP comparison of current vs. preliminary gene models. Most of the updated gene models (84.0%) match the preliminary models completely. An additional 14% of the updated gene models have best matches to preliminary models with Genbank IDs that are either identical or numerically adjacent (indicating that the same open reading frame is under consideration, but a gene split occurred during manual annotation). These updated models differ from their corresponding preliminary models to varying degrees in length (either longer or shorter) and/or amino acid sequence (see Additional file 5). 103 models have no significant match (E < 0.001) among the preliminary set, but it is notable that these include an atypically high number of shorter peptide sequences (median length = 146 amino acid residues), and that all but 21 of these 103 models are annotated only as "hypothetical proteins", suggesting the presence of spurious predictions among them. The remaining 1% of the updated models have a best match to a preliminary model with a non-adjacent Genbank ID (e.g. a related gene family member or a domain match), indicating that the original models have undergone substantial structural modifications.

### Selenocysteine-containing genes

As reported previously [3,74], *T. thermophila *expresses a tRNA predicted to decode the only *Tetrahymena *stop codon, UGA, into selenocysteine (Sec). Sec, the so-called 21^st ^amino acid, is found in all three domains of life, often at the active site of certain redox enzymes [75,76]. In eukaryotes, translation of UGA to Sec depends on the presence of a conserved secondary structure, known as the Selenocysteine Insertion Sequence (SECIS), in the 3' untranslated region of the mRNA [77]. Because UGA is only rarely translated as an amino acid codon, standard gene prediction programs cannot accurately annotate selenoproteins.

We searched the genome and predicted proteome of *T. thermophila *for homologs of known Sec-containing proteins from humans [78], *Drosophila *[79], *Chlamydomonas *[80], *C. elegans *[81], *P. falciparum *[82], and *Emiliania huxleyi *[83] using BlastP against predicted *Tetrahymena *proteins and TBlastN against the whole genome assembly. No significant matches were detected for a number of the query proteins, including the four *Plasmodium *proteins, which are so far confined to the apicomplexans. Two of these four have been predicted to be targeted to the apicoplast [82], an organelle for which we and others have failed to find evidence within the ciliate lineage [3,5].

Meaningful alignments in the regions of known Sec residues from other organisms were examined manually for signs of misannotation of exon/intron boundaries and/or stop sites that might have resulted from an unrecognized in-frame UGA. Six cases of putative *T. thermophila *selenoprotein-encoding genes were identified (Table 2). In the course of inspection, structural annotation of three other genes was manually improved in the region of interest without involving a putative Sec codon (Table 2). Based on this evidence, the structural annotation of these nine genes was updated in the most recent Genbank submission. The six predicted putative selenoproteins include two glutathione peroxidases (GSHPx), three members of the thioredoxin reductase (TrxR) family, and one homolog of selenophosphate synthetase 2. The first two enzyme families are associated with cellular defense against oxidative stress and, not surprisingly, *Tetrahymena *ESTs from nearly all genes in these families are found disproportionately in libraries produced from stressed cells, in particular starved and heavy metal-treated cells (see Table 2 and Additional file 3). The putative selenoprotein-encoding gene TTHERM_00047660 is an exception to this trend, with ESTs being abundantly represented in vegetatively growing cells, suggestive of a role in non-stress cell metabolism.

The predicted 3' UTR sequence of each *Tetrahymena *homolog of a selenoprotein from the species described above was tested for the presence of a SECIS using SECISearch [78,84]. The six putative selenoproteins, and only those six, were positive (see Table 2 for the RNA sequences predicted to form the secondary structures). It is quite possible that *T. thermophila *contains other selenoproteins, not readily detectable as homologs of known selenoproteins in other organisms, that may be detected by a more systematic search for SECIS elements downstream of predicted genes (as in [82]). Additional comparative genomic information from related *Tetrahymena *species will aid in the verification of such candidates.

### Condition-Specific EST Representation

We examined the frequencies at which genes were hit by ESTs from each of the six conditions as potential signs of differential gene expression. Additional file 3 is an Excel spreadsheet of all 9,709 predicted protein-coding genes hit by one or more EST, with the number of EST hits broken down by cell condition. Generally, genes predicted to be highly expressed in a given condition were indeed found to be over-represented among the ESTs from that condition. For example, the top gene in conjugation EST abundance is *ngo*A, an apparently non-protein-coding gene of unknown function that is also induced during starvation [53,58]. Two of the next three (found in no other condition) are *TWI*1 and *PDD*1, both implicated in conjugation-specific genome rearrangement [51,54]. *PDD*1 encodes a chromodomain protein that acts through binding of lysine-methylated histone H3 [85,86]. Another uncharacterized chromodomain protein-encoding gene is among the top conjugation ESTs, as well as a gene encoding a protein containing a *jmj*C domain, which has recently been implicated in the demethylation of histones [87]. Methylation of histone lysine residues is carried out by SET domain-containing proteins [88], and several genes encoding these are over-represented by conjugation ESTs. These examples, as well as numerous genes annotated as "hypothetical" found to be differentially represented among the conjugation EST set, are promising candidates for experimental analysis, using the tools of *Tetrahymena *reverse genetics [1,89].

All five metallothionein genes [47] are among the ESTs found predominantly in the heavy metal cDNA library, as are other genes with potential roles in response to oxidative stress (e.g. thioredoxins, glutathione S-transferases and peroxidases). A number of the genes over-represented among starvation ESTs encode proteases and proteasome subunits, which may be required for scavenging nutrients from both extracellular and intracellular sources [90,91]. A more quantitative assessment of genome-wide differential gene expression will be facilitated by interrogations of whole genome expression microarrays, which are in progress (W. Miao, M. Gorovsky, et al.; pers. comm.).

**Figure 3:**
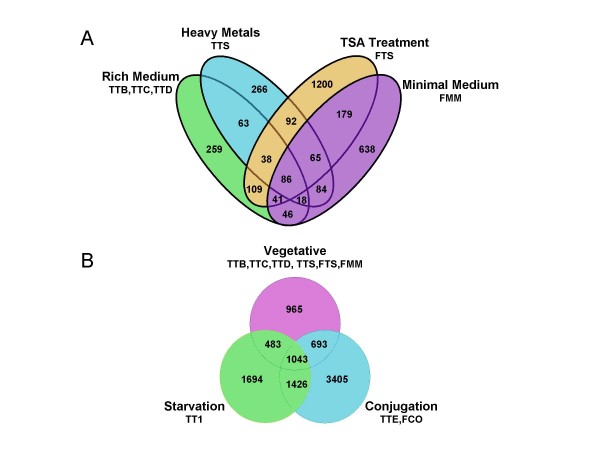
**Venn diagrams of the overlap in EST representation for all genes detected in (A) the four vegetative growth conditions and (B) the combined vegetative pool vs. starvation or conjugation**.

The overlaps between genes represented in the EST pools are displayed in the Venn diagrams of Figure 3. Figure 3A shows the overlap between the four log phase, vegetative growth conditions (rich medium, rich medium plus heavy metals, rich medium plus TSA, minimal medium). As the diagram reveals, there is considerable diversity in the EST representations. The four most numerically abundant gene subsets are those unique to each of the four conditions while only 86 predicted genes are hit by one or more EST in all four conditions. However, much of the apparent lack of overlap is due to undersampling of the EST libraries. Of the 2,363 genes hit only by ESTs from a unique growth condition, 80% were hit by only a single EST, and only 5% were hit by four or more. In Figure 3B, the four vegetative conditions are lumped together and compared for overlap with the conjugation and starvation conditions. Again, the three most abundant subsets are those unique to each of the conditions. Of the 6,064 genes in these unique subsets, 54% are represented by only a single EST, while only 14% are represented by four or more.

## Conclusion

We report here substantial progress in finishing and reannotating the MAC genome sequence of *Tetrahymena thermophila*. These results represent the culmination of the concerted effort begun in 2003. Additional refinement of these genomic resources, as described below, is highly desirable and will be sought in the near future, but the current picture is greatly enhanced relative to our first report [3]. Over 60% of the genome is essentially finished, and the vast majority of MIC contamination has been removed from the assembly. To this point in the finishing phase, no exceptional difficulties have been encountered in closing sequencing gaps due to, e.g., complex repetitive regions. In addition, all physical gaps examined thus far have been small (< 500 bp). Ongoing HAPPY mapping has now linked over 97% of the MAC genome into super-scaffolds. We therefore predict that, with a limited additional closure effort in targeted regions, the entire *T. thermophila *MAC genome sequence can be finished, which would be a remarkable achievement for a genome of this size. The updated MAC genome sequence presented here will be a powerful tool for understanding eukaryotic chromosome structure, and an indispensable guide for the assembly of the germline MIC genome (sequencing of which is in progress; ), which will be a greater challenge due to its abundance of repetitive sequences.

By a combination of EST sequencing and analysis, improved gene finding, and manual curation, we have addressed many of the shortcomings of the initial *T. thermophila *structural annotation. In addition, we have gained new insights into *Tetrahymena *biology through the detection of alternative splice sites previously unknown in this genome, the identification of selenoprotein-encoding genes, and the analysis of patterns of differential EST abundance. The updated set of 24,725 gene models will provide a new framework for conducting and interpreting *Tetrahymena *genomic and post-genomic research, as well as for comparative genomic studies involving the ciliates. Further improvements to structural annotation (especially of genes expressed at low abundance) will benefit greatly from the comparison of sequences conserved between *T. thermophila *and related species within the *Tetrahymena *genus and from generation of full-length cDNA sequences.

## Abbreviations

CGH: Comparative Genomic Hybridization; EST: Expressed Sequence Tag; E value: Expect value; HAPPY: HAPloid PolYmerase chain reaction; IES: Internal Eliminated Sequence; MAC: Macronucleus/ar; MIC: Micronucleus/ar; maD: MAC-destined; miL: MIC-limited; PASA: Program to Assemble Spliced Alignments; RT-PCR: reverse transcriptase polymerase chain reaction; SECIS: Selenocysteine Insertion Sequence; TSA: Trichostatin A.

## Authors' contributions

RSC coordinated EST sequencing and analysis, provided minimal medium-grown cells and analysis of the corresponding ESTs, and prepared the manuscript. MT conducted bioinformatic analyses on EST sequences, performed manual and automatic gene model curation, coordinated Genbank submissions, and generated figures and tables for the manuscript. KMJ and LJT coordinated genome closure efforts. JRW coordinated reannotation efforts. BJH assisted in applying PASA to EST-to-genome alignment. DMC provided heavy metal-treated cells and analysis of the corresponding ESTs. EAW and JJS provided TSA-treated cells and analysis of the corresponding ESTs. KC and SRL provided starved cell RNA and analysis of the corresponding ESTs. MTC performed RT-PCR to confirm alternative splicing. YL provided conjugating cells and analysis of the corresponding ESTs. JG and REP coordinated cDNA library construction and TBestDB data submission. EPH and EO designed, conducted, and analyzed the comparative genomic hybridization experiments and assisted in preparing the manuscript. JAE and BAM provided oversight of the project and assisted in data analysis and preparing the manuscript. All authors read and approved the final manuscript.

## Supplementary Material

Additional file 1 **Normalized MIC and MAC array hybridization ratios**. Values are log_2 _MIC or MAC hybridization relative to a MAC reference. The average over all probes within each scaffold is given for each separate array hybridization (see Methods for probe details), then the average of the three MAC hybridizations and of the two MIC hybridizations. Scaffolds with MIC ratio averages greater than 1.3 were designated MIC-limited (miL); others were designated MAC-destined (maD). For each scaffold with a tblastn hit (E < 0.001) to a known *T. thermophila *MIC-limited transposon gene, the identity and E value of the best hit is given. Click here for file

Additional file 2 **Summary of genome closure progress**. Shaded rows contain two or more component scaffolds that were joined to create larger scaffolds. The first accession number (in italics) corresponds to the largest component, which is retained by Genbank as the number of the newly merged scaffold. A. Fully finished complete chromosomes (telomere-capped at both ends). B. Fully finished single-capped scaffolds. C. Complete chromosomes, not fully finished, exceptions noted. D. Single-capped chromosomes, not fully finished. Click here for file

Additional file 3 **EST to gene mapping**. List of all predicted protein-coding genes hit by one or more EST, with the number of EST hits broken down by cell condition. Abbreviations are as in Table 1. ALL VEG represents the sum of ESTs from the four vegetative growth conditions. EC Numbers, Gene Ontology (GO) Terms, and Common Names were generated by an automated annotation process. The column "PASA Assemblies" indicates the number of assemblies that map to each predicted gene. Click here for file

Additional file 4 **Putative cases of alternative splicing**. A. Summary table. B. Alternate protein-coding sequence potential and splice junctions. Each pair of sequences is preceded by the corresponding gene model ID. The accepted Genbank model protein translation of each gene is followed by the alternate translation. In each case, attention is drawn to the differences between the two sequences by underlining amino acid residues added, or those at the junction of an addition in its corresponding partner. Residues at the junction may not match the partner due to splitting of codons by mRNA splicing. Predicted protein sequences are followed by a portion of the corresponding genomic DNA region. Relevant GTAG splice junctions are underlined. Click here for file

Additional file 5 **BlastP comparison of current vs. preliminary gene models**. Each predicted peptide sequence of the updated gene model set (query) was blasted (wublastp) against the complete set of preliminary predicted peptide sequences (target). The top match (E < 0.001) to each is given, as well as statistics on the range of aligned sequence and percent identity. The data are sorted, in descending order, by Percent Identity, then by Percent of Query Sequence Aligned, then by Percent of Target Sequence Aligned. Click here for file
