# Supplementary material for: Refined annotation and assembly of the Tetrahymena thermophila genome sequence through EST analysis, comparative genomic hybridization, and targeted gap closure
Source: BMC Genomics. 2008 Nov 26;9:562. doi: 10.1186/1471-2164-9-562 (PMC2612030; doi:10.1186/1471-2164-9-562)
Supplement: Additional file 4 — Putative cases of alternative splicing. A. Summary table. B. Alternate protein-coding sequence potential and splice junctions. Each pair of sequences is preceded by the corresponding gene model ID. The accepted Genbank model protein translation of each gene is followed by the alternate translation. In each case, attention is drawn to the differences between the two sequences by underlining amino acid residues added, or those at the junction of an addition in its corresponding partner. Residues at the junction may not match the partner due to splitting of codons by mRNA splicing. Predicted protein sequences are followed by a portion of the corresponding genomic DNA region. Relevant GTAG splice junctions are underlined. [file 1471-2164-9-562-S4.doc]

**A.**

| **Gene ID** | **Nature of alternative splicing** | **RT-PCR Confirmation** | **Protein Features** |
| --- | --- | --- | --- |
| TTHERM_00008690 | Intron removal/non-removal | Y | hypothetical |
| TTHERM_00191720 | Alternate 5’ splice junction | Y | Zn-finger |
| TTHERM_00241790 | Alternate 3’ splice junction | Y | Ca-transporter |
| TTHERM_00492860 | Alternate 5’ splice junction | Y | hypothetical |
| TTHERM_00455430 | Intron removal/non-removal | Y | Protein kinase |
| TTHERM_00459230 | Intron removal/non-removal | No product | RAD51-related |
| TTHERM_00522030 | Intron removal/non-removal | Y | Basic Leucine zipper |
| TTHERM_00476650 | Intron removal/non-removal | Y | PHD Zn-finger |
| TTHERM_00418290 | Intron removal/non-removal | N.T. | aminotransferase |
| TTHERM_00637820 | Alternate 3’ splice junction | Y | SNARE domain |

**B.**

TTHERM_00008690

model 3 ESTs Intron retention

MDIQENQSQQVEKQESLQKQDENVDKLKQIRASIKSMEVKQIKELRRLLRIENLQNVEEITTLEYLMMIMNNNALRSALVQLQNQYKMMYRTLGDGNCFYRSIMLNKVFWLVEQKSCEPLEQFIIQINNIPQLQTIFQTVPINANSLKSIYLLFLFKILEDKKSNKQLRMLDIIRMYNSLPEVDFASVIICRQMIFNTYESFKVHDSFKDFLDDDMKEKIEFVLLRYFYEAQYVIIPLAAQTFQCELNIQNFYQPDQYSSKVNIEKLNYSPIEVENKDAQMPSFNLIYNGGHYELAILDSQAKTDFYKINKQMISFNTQYSDQQYYYFIQNFIQRYQTEIIRMNNIEESAFYNQQNGSNEEEEYQTLDWQKLQRSEEYRRQNYENQKLKVLIENLQNENNQVKQQDDKLQQKQQSNSQIDSQQEKNSKNQQNEIPIELDEKNQVNKTNQYQPKIQQKSQSSQFDQNNQNLQFQNLPSKNSMTNQQNNSGENDVNNMKYDQQDQRVLDFQNMKRICVQCNKKLPSDFKYPFFVLPMDEDIPDNVVLRICTDCFIDLASKYQNDQEFIEVNGKLYQLSPRLIGELISFIQQSSKKNQKN*

alternate 2 ESTs Intron removal

MDIQENQSQQVEKQESLQKQDENVDKLKQIRASIKSMEVKQIKELRRLLRIENLQNVEEITTLEYLMMIMNNNALRSALVQLQNQYKMMYRTLGDGNCFYRSIMLNKVFWLVEQKSCEPLEQFIIQINNIPQLQTIFQTVPINANSLKSIYLLFLFKILEDKKSNKQLRMLDIIRMYNSLPEVDFASVIICRQMIFNTYESFKVHDSFKDFLDDDMKEKIEFVLLRYFYEAQYVIIPLAAQTFQCELNIQNFYQPDQYSSKVNIEKLNYSPIEVENKDAQMPSFNLIYNGGHYELAILDSQAKTDFYKINKQMISFNTQYSDQQYYYFIQNFIQRYQTEIIRMNNIEESAFYNQQNGSNEEEEYQTLDWQKLQRSEEYRRQNYENQKLKVLIENLQNENNQVKQQDDKLQQKQQSNSQIDSQQEKNSKNQQNEIPIELDEKNQFQNLPSKNSMTNQQNNSGENDVNNMKYDQQDQRVLDFQNMKRICVQCNKKLPSDFKYPFFVLPMDEDIPDNVVLRICTDCFIDLASKYQNDQEFIEVNGKLYQLSPRLIGELISFIQQSSKKNQKN*

>CH445775

GATAGAATTCGTACTGTTAAGATATTTCTATGAAGCTCAATATGTAATTATTCCCTTGGCTGCTCAGGTAAAATTTTGTGTTTATTAACTTTTATTTATAAAACTTTTTATATTTAATAGACGTTTTAATGCGAGCTTAATATTTAGAATTTTTATTAGCCTGATTAGTATTCAAGCAAAGTAAATATCGAAAAGCTGAATTACTCACCAATCGAAGTAGAAAATAAAGATGCTTAAATGCCCAGTTTCAACCTTATTTATAATGGTGGACATTATGAGCTAGCTATTTTGGATTCTTAAGCTAAAACAGACTTTTACAAAATAAATAAACAAATGATATCATTCAACACTCAATATTCAGACTAGCAATATTATTATTTCATATAAAACTTTATATAAAGATATCAAACAGAAATTATAAGAATGAATAACATAGAAGAAAGTGCTTTCTACAATTAATAAAATGGCTCTAACGAAGAAGAAGAATATTAAACTCTAGATTGGTAAAAATTGCAAAGAAGTGAAGAATACAGAAGATAGAATTATGAAAATTAAAAGTTGAAAGTATTAATTGAAAATTTGTAAAATGAAAACAATTAGGTGAAACAATAAGACGACAAGCTACAACAAAAGCAATAGTCGAACTCTCAAATAGATTCTTAATAAGAAAAAAACTCTAAAAACTAATAAAATGAAATACCTATTGAACTAGATGAAAAAAATTAAGTAAATAAAACTAATTAATATTAACCAAAAATTTAACAAAAGTCATAATCTTCTTAATTTGATTAAAACAATTAAAATTTATAGTTTCAAAATCTTCCTTCAAAAAATTCTATGACCAATTAATAGAATAACTCAGGAGAAAATGACGTTAATAATATGAAATATGACTAATAAGATCAGCGCGTATTAGACTTTTAAAACATGAAAAGAATATGTGTGTAGTGTAATAAAAAACTTCCATCCGATTTT

TTHERM_00191720

model 5 ESTs

MNPSQMNMNPMGMPQNMNSGMMPQGGQMNSQGMMQHQHYQNNGQRNMQQNNMNNRGGNRGNGGQNGGGYKGGNQRDNYGGQGGNSRFGGNNQYVGSGSGKFSSQHQQQKGQQNAPPMVRRPIQHNNRLGQGGRPGGPSGQPPRRDMRGPKNEVSVTAFHKTKMCPTVEAGQQCKKGDKCGYAHTQVELREPPNLKKTKLCQLFKTTRCNKGDSCDFAHGTEELKSYVDRYKTQICQQFTQKGSCQNGDKCHYAHGEQEIRQPQFSHQSQPQQVPQGQVQGGQLQPSQGAHMSLAHQQQGQQVAGTTAAYQQQMNPSQIPYGYTDQSGLYANNTQYVQYAQQGAATTAYGQAQPVQQGLYTQPLPQTQVYTQSYPTSTLQQYTSQQPPIQYFQQPQQYQSYV*

alternate 6 ESTs Alternate 5’ splice junction

MNPSQMNMNPMGMPQNMNSGMMPQGGQMNSQGMMQHQHYQNNGQRNMQQNNMNNRGGNRGNGGQNGGGYKGGNQRDNYGGQGGNSRFGGNNQYVGSGSGKFSSQHQQQKGQQNAPPMVRRPIQHNNRLGQGGRPGGPSGQPPRRDMRGPKNEGQDNQSYQNNQQGFQNGSRGGPKKQSSGVQKNKNHSHKQDINSNKLSVTAFHKTKMCPTVEAGQQCKKGDKCGYAHTQVELREPPNLKKTKLCQLFKTTRCNKGDSCDFAHGTEELKSYVDRYKTQICQQFTQKGSCQNGDKCHYAHGEQEIRQPQFSHQSQPQQVPQGQVQGGQLQPSQGAHMSLAHQQQGQQVAGTTAAYQQQMNPSQIPYGYTDQSGLYANNTQYVQYAQQGAATTAYGQAQPVQQGLYTQPLPQTQVYTQSYPTSTLQQYTSQQPPIQYFQQPQQYQSYV*

>CH445644

AGAATTACATAAATCAAGAAAGTTATTGATATTTACTAGTTTTGTTATAAATACTTAATCAAATATGAATCCATCACAAATGAATATGAATCCCATGGGTATGCCCTAGAATATGAATTCTGGTATGATGCCTTAAGGAGGTTAAATGAATTCTTAAGGAATGATGCAACATTAACATTACTAAAATAATGGATAAAGAAACATGCAATAAAATAATATGAATAATGTAAGATTTTATTGTTATAGATATACTTAGTATTAAATATTAAACATGTAAAAAAATAGAGAGGCGGCAACAGAGGAAACGGCGGCTAAAACGGTGGAGGTTACAAAGGTGGAAACTAAAGAGGTAATTAGAATTGCACATAATTTATTTAGTCTTTACTTAGAAAGAAAGACATATTTATTTAAGTGTCTCCTTCAAAAATTAAATAAAATTTTGAATATTTTTAGATAACTATGGAGGCTAAGGTGGAAATAGTAGATTTGGAGGAAACAATTAATATGTTGGAAGTGGCAGTGGAAAGTTTAGCAGCTAGCATTAATAATAAAAGGGATAATAAAATGTACATTTTTGAAAAATTTTTAATAATTTATTAAATAGTGTCTAATTTATTTAATTTTTTTGAAAGTTAGTCAGTCTATTGAGATGTGACTACTTAATTAACCTTTACTCTATTTTAAACATAAATAAATCTTTATAAAAAGAAAAAAACATTTATTAGAAAAATTTTCAATTATTTTATTAACAGGCACCACCTATGGTGAGAAGACCCATTCAACACAATAACCGTTTAGGACAAGGAGGCAGACCTGGCGGACCAAGCGGATAACCCCCCAGAAGAGATATGCGTGGGCCCAAAAATGAAGGTTAGGACAATTAAAGTTATTAAAATAATTAATAAGGATTCTAAAATGGGTCTCGTGGTGGACCCAAGAAACAAAGCAGTGGTGTATAAAAAAATAAAAACCACAGCCATAAATAAGACATAAATAGCAATAAATGTATTTGCTTATTTAATTAATTAATAAACCATTTTTTAAAAGCAGTCATTAAATAATTTAAATAAAAACAGTAAGTGTGACAGCTTTCCATAAAACCAAAATGTGTCCTACAGTAGAAGCCGGTCAATAATGTAAGAAAGGAGATAAGTGTGGATATGCTCATACATAGGTTGAATTAAGAGAGCCTCCTAACCTTAAAAAAACAAAGCTTTGCTAACTCTTTAAAACAAGTAATTAAAATTAATTAGCATATTTATTAGTTCATATATAATAAAATATTATTAATAAATAGCACGTTGCAACAAGGGAGATTCATGTGATTTTGCACATGGTACTGAAGAACTTAAAAGTTATGTTGACCGCTATAAAACACAAATTTGCTAGTAATTTACATAAAAAGGTTCTTGCTAAAATGGTGACAAGTGTCACTATGCTCATGGAGAACAAGAAATCAGATAACCGTAAATTAATTAATTATTTTAAGCTTAAATATATTAAGATTCAAAGTTGATCTCCAAAATCTGGTATTGTATTCCCAATGCAATCAATCAATCAGTCATTCATTAAATCTATAAATCAACTTGATAGCCAAAAAATGAATTGCAAAAAAATATTTAAAAACATTATTGTTAATAAATTTAGATAATTTTCTCATTAGAGTTAGCCATAGTAGGTTCCATAAGGCCAGGTTTAAGG

TTHERM_00241790

model 2 ESTs

MDHTIQISTTEQSSQELVPKRDIDDIFEIYQDRKVTDFKEYKNLDGLLEKLNVNKATGLDHHNLDDLQKRRSMYSVIVDQEDKSKSIFEYIFDALEDVMLRLLIVVACISICLGIIQDGWAKGWFEGTSIIISVIIVVSIQSYSDYSKEKQFAVLHKIQSVQEHETKRNGHIHMLNVKDWVVGDIIQITPGCVPKADGILINCDIPIQTDESSLTGENEPQHKEIGCCIFEGCPIVDGYGEMVVLRLGSDSCQGRIKALMSEEDEEETTPLQDKLEIMANQIGVLALYAASITFITLISFQIYNNYKKDLCFFCLEFFQDLSRHLMAAFGIIIMAIPEGLPLSVTIALAYSVHQMYLEKNLVKKLKSCETMGGVSDICTDKTGTLTYGNMILKKILLNSNSYNVESLVNNKSQTKELLLKIIQNTSKAIVDVSDDGVAIQKGNITEIGLIKWILEQEQPRSYYNQQKIREFPFTSKNRCSGTIVNVNGTQYLYVKGQPDTIIPSCSNVYHNDEIIEFDEAKKQKILKNIEEHNCLAFRGLTFAVKQIEGDFSSDFNTVQLNDLISSNDLTFVATCYLQDIVREEVPKAVESLRKAGVTTRMITGDSYQTAKAIGIQTGIIYPHEESAITTGYQIAQMSELELSSCVDKFKIVAKCNPEQKLKFVKALKSQDKIVAVTGDGTNDALCFQVSDVSFTMGQKGTEIIKEAGDIILIDDNYASIVTACSWGRNIQEGIRKFLVFQLTVNIVGVFICLLGSIIIQESPLSSSQMLWINLIMDTFASLALATDHPTEELLRRQPYRRNEDLINGYMKRNIVFQCIYQIIALSFVLLYGDNIFGVPEMQYLDVHEFNHNGAIHMTIFFHTFVLLQLFNEFNCRDLRRDILNPFKDIFRNRYFLGVIIFSFIVQYSLVFFGGQTFRCTRISLTQHLFCIIIGMGGLLVGLIVNLIPESFYESIAMFRDYNQLQIYESQRKYSACQVNETYQHDEGEYGSMSMEHQENIKKELFGEKNL*

alternate 5 ESTs Alternate 3’ splice junction

MDHTIQISTTEQSSQELVPKRDIDDIFEIYQDRKVTDFKEYKNLDGLLEKLNVNKATGLDHHNLDDLQKRRSMYSVIVDQEDKSKSIFEYIFDALEDVMLRLLIVVACISICLGIIQDGWAKGWFEGTSIIISVIIVVSIQSYSDYSKEKQFAVLHKIQSVQEHETKRNGHIHMLNVKDWVVGDIIQITPGCVPKADGILINCDIPIQTDESSLTGENEPQHKEIGCCIFEGCPIVDGYGEMVVLRLGSDSCQGRIKALMSEEDEEETTPLQDKLEIMANQIGVLALYAASITFITLISFQIYNNYKKDLCFFCLEFFQDLSRHLMAAFGIIIMAIPEGLPLSVTIALAYSVHQMYLEKNLVKKLKSCETMGGVSDICTDKTGTLTYGNMILKKILLNSNSYNVESLVNNKSQTKELLLKIIQNTSKAIVDVSDDGVAIQKGNITEIGLIKWILEQEQPRSYYNQQKIREFPFTSKNRCSGTIVNVNGTQYLYVKGQPDTIIPSCSNVYHNDEIIEFDEAKKQKILKNIEEHNCLAFRGLTFAVKQIEGDFSSDFNTVQLNDLISSNDLTFVATCYLQDIVREEVPKAVESLRKAGVTTRMITGDSYQTAKAIGIQTGIIYPHEESAITTGYQIAQMSELELSSCVDKFKIVAKCNPEQKLKFVKALKSQDKIVAVTGDGTNDALCFQVSDVSFTMGQKGTEIIKEAGDIILIDDNYASIVTACSWGRNIQEGIRKFLVFQLTVNIVGVFICLLGSIIIQESPLSSSQMLWINLIMDTFASLALATDHPTEELLRRQPYRRNEDLINGYMKRNIVFQCIYQIIALSFVLLYGDNIFGVPEMQYLDVHEFNHNGAIHMTIFFHTFVLLQLFNEFNCRDLRRDILNPFKDIFRNRYFLGVIIFSFIVQYSLVFFGGQTFRCTRISLTQHLFCIIIGMGGLLVGLIVNLIPESFYESIAMFRDYNQLQIYESQRKQSEEHLLNIDSACQVNETYQHDEGEYGSMSMEHQENIKKELFGEKNL*

>CH445533rev

TTATCTTCCAGCTAAATGCTTTGGATTAACTTGATTATGGATACTTTTGCAAGTCTCGCTTTGGCAACTGATCATCCCACTGAGGAACTTTTAAGAAGACAACCTTATAGAAGAAATGAAGATTTGATCAATGGTTACATGAAACGTAATATTGTCTTCTAATGCATTTACTAGATTATTGCTTTATCTTTTGTCCTTTTGTATGGTGACAACATATTTGGTGTCCCAGAAATGTAATACTTGGATGTTCACGAATTCAATCATAATGGAGCTATACACATGACAATCTTTTTCCATACTTTCGTTTTACTTTAACTTTTCAACGAATTCAACTGCAGAGATCTTCGTAGAGATATACTTAATCCCTTTAAGGATATCTTCCGTAACAGATATTTCTTAGGTGTTATCATTTTCAGCTTTATAGTCTAGTATTCTTTAGTCTTTTTCGGTGGTTAAACTTTCAGATGCACTCGTATCAGCTTAACCTAACACCTTTTCTGCATCATTATCGGTATGGGTGGTCTATTAGTTGGACTAATCGTTAACCTTATACCTGAATCATTCTATGAATCAATTGCTATGTTCAGAGATTACAATCAACTTTAGATTTATGAAAGTCAAAGAAAATGTAAAATAAATATCTTTTAATTGTTTATCATCAATTTATTTCTATTTATGAAAATTTATTTTAGAATCTGAAGAGCATTTATTAAATATAGATTCAGCTTGCCAAGTAAATGAAACATACTAACACGATGAAGGTGAATATGGTAGTATGTCTATGGAACACTAAGAAAACATCAAAAAAGAATTATTCGGGGAAAAGAATCTCTGAATAATTAAGTAGTGAAAATTAAAATATCGACTTAACTAAAAATGAAGCAAAAAACTCTACTTCAAATGACCAGCAGATTGGTTGCTAAAGACATTCCCAAAATTTGTTAGCAAAACAAATAGAAAAAATGTTTTTAAATATCAGTCTAGCTATTTATATTTTTATTATAATTATAAAATAATTGA

TTHERM_00492860

model 2 ESTs

MQQDGDYQMELQSSSSRRRQRSLNSSKSKRRDLKRSKSCKNFDQICKHTFTFRQLSEEINGLLPLEVTIKMCPQKQKIYDGSTEKLNTFPVIIIDEKPHMPLFVNECEIKAYMEHEEELLQNYEEKRRIQIKPSFGDHAPKEQFCSVCMVKYENSYLEHIQSVQHKSKYRQNQYAKIICDLELEWKKQKQDLDHSSNAKNNQMEDFENLKTLVDSEQSNQIKNAKLFNEPQKNLFEDQEKNIGENNQNIWNLQQVNLIQSNNKLQKCNTDQSNKKIFNDQSFNTNKRMAGSLSYAKFQNPVNSAFRKYNLSTQPDEQFIDESQQLRQQQFTYQSIKNISIVSIFIYLQKRNMQSEKFEDGSMAKDNCKQIKQTNKQQKEEQQKKGFQVNKSFFKEHLLYNHLKQKQIALELQQKQEDMKENNLNFCSNQESILKLGYENQVSSDMDSNRSAKNIETKGQSSSAQKFVQRNNQHQKDSQLLQISRASSSSCSRKNQSHSKSCNGGYKFSFDEIQSFQAKEAEGQISQQESKLPDTKQKEHNYQPISKNKKRSFQEYNYQRGMQMSCEEEISINKKFKIFGIGTTEIKSIWQQFKDIFALQFTGVKTKQNN*

alternate 2 ESTs Alternate 5’ splice junction

MQQDGDYQMELQSSSSRRRQRSLNSSKSKRRDLKRSKSCKNFDQICKHTFTFRQLSEEINGLLPLEVTIKMCPQKQKIYDGSTEKLNTFPVIIIDEKPHMPLFVNECEIKAYMEHEEELLQNYEEKRRIQIKPSFGDHAPKEQFCSVCMVKYENSYLEHIQSVQHKSKYRQNQYAKIICDLELEWKKQKQDLDHSSNAKNNQMEDFENLKTLVDSEQSNQIKNAKLFNEPQKNLFEDQEKVQDQFQEKILITVKSNIGENNQNIWNLQQVNLIQSNNKLQKCNTDQSNKKIFNDQSFNTNKRMAGSLSYAKFQNPVNSAFRKYNLSTQPDEQFIDESQQLRQQQFTYQSIKNISIVSIFIYLQKRNMQSEKFEDGSMAKDNCKQIKQTNKQQKEEQQKKGFQVNKSFFKEHLLYNHLKQKQIALELQQKQEDMKENNLNFCSNQESILKLGYENQVSSDMDSNRSAKNIETKGQSSSAQKFVQRNNQHQKDSQLLQISRASSSSCSRKNQSHSKSCNGGYKFSFDEIQSFQAKEAEGQISQQESKLPDTKQKEHNYQPISKNKKRSFQEYNYQRGMQMSCEEEISINKKFKIFGIGTTEIKSIWQQFKDIFALQFTGVKTKQNN*

>CH445759

TGTCTGAGGAAATAAATGGGCTACTACCATTAGAAGTTACTATTAAAATGTGTCCCTAAAAATAAAAGATATATGATGGATCAACGTAAAGAAAAAATAACTAAATATATATAATAAAAATAACATTTGTCTATCAAAAGTCTTTAGAAATTTATGCCATTTATTTTTAAATTTTATAATTATTTTAGAGAAAAGCTTAACACTTTTCCTGTGATAATAATTGATGAAAAACCTCATATGCCTCTCTTTGTAAATGAGTGTGAAATAGTAATACTAAAATATGAAGCTATAGCAGAAAACACATTCATTATATTTTAATTTTATGATTAAATAGAAAGCCTATATGGAGCATGAAGAAGAGTTGTTACAAAACTACGAAGAAAAGAGAAGAATATAAATTAAGCCTTCATTTGTAAGTGAATTATGGATCTCTAAATTCTTTTCTTTCAATTTTTCTAATTTACAAAAGCAAAACCCTTTGGAGATAAATTCAATTGAAATGGAAAACAATAATTTGAATTTTTTTAAGTTCGATTTTCAAAAGAATTAAATTGCTCTGAGCATAAAGTTCAAGTTTAATGCAAGTGCTAAATGCATTTAAAAAAGAGTTTATAAATAAGTTTAGTAGCTCAGCTATTTAAAAGCATATTTATATAATTATATTTTAAATTTTTAGGCAAAATTTGCTTCAATTTAATAAAATTATTTTAGCTTGAAATTTGGTTTAGCATTTTATGAGATTTTGAAAATAATTCTTTGATATTTACAGGGAGATCATGCACCTAAAGAATAATTTTGTAGTGTCTGTATGGTTAAATATGAAAATTCTTATTTGGAGGTAAAATTAACTTAAAATTTAAGTAATTTAGTTAGACACACTTTTAATTAAAATAGCATATCCAATCTGTATAACACAAAAGCAAATATAGACAAAATCAGTATGCCAAAATTATTTGTGATTTAGAATTAGAATGGAAAAAGTAAAAGCAAGATTTAGATCATTCTTCAAATGCTAAGAACAATCAAATGGAAGATTTTGAAAACCTCAAAACTTTAGTTGATTCTGAGTAGAGCAACTAGATCAAGAATGCAAAACTTTTTAATGAGCCCTAAAAAAATTTATTTGAAGATTAGGAAAAGGTATAAGATTAATTTCAAGAGAAAATTCTGATTACTGTTAAATCTGTAATCATTTTATTTAATGTTACTATATTAATATTTTAACATTTTTAATAAAATATGAAAATTCTTAGAATATTGGAGAAAACAACTAAAATATTTGGAATTTACAATAAGTCAATTTAATCTAATCAAACAATAAGCTATAGAAATGCAATACAGATTAAAGCAATAAAAAGATTTTTAATGATCAATCATTTAACACTAATAAACGAATGGCAGGCAGTCTTTCCTATGCTAAATTTTAAAACCCAGTAAATTCTGCATTTAGAAAATACAATTTATCTACCTAGCCAGATGAGCAATTTATTGATGAAAGTTAATAGCTTAGACAATAATAGTTCACATACCAAAGCATAAAGAATATATGTAAAACACTTAAATTTGAAGAAAAGTAATTTTATAATTTCTATTTAAAGCGATTGTAAGTATATTTATTTATTTATAAAAAAGAAATATGTAAAGTGAAAAATTTGAAGATGGATCGATGGCTAAAGATAATTGCAAATAGATAAAATAAA

TTHERM_00455430

model 1 EST Intron removal

MGNSLCMEQKVEKNQQKTKKSGLQSSNNDLNEYKLPADPKEKKLFQSQAGNHSLGEEEKITLSSFRIIRVIGSGAFGKVYLVNKKGTEEVYAMKVYDKMTLYEKNVILSTIGERNILKNMNSNFIVKLHYAFQAAQNLYLMMDFMVGGELYYHLKKAKKFDEERTKFYIAQVILAIECLHQNNIMYRDLKLENILLGQDGYIKLTDFGLSKEGIKDKDLTNTLCGTAEYMAPEQIMNEGHNKMADFWQIGVLTYEMLYGTTPFFNEFRPNKDEIFNDIIQGKYTFPQYFKDDAKNFIRGLLQKDVKKRLGYNGFHELKKHAFFADFDWDKLERQEIEPQFKPPLRSQVDLQNFNPRLVKNTMTDLGVKDNKPHLKEEIESQFRGFTFVKDDHLNTDVPQRNSSAERNN*

alternate 3 ESTs Intron retention

MGNSLCMEQKVEKNQQKTKKSGLQSSNNGNNKNQRKNERLNSIIDLNEYKLPADPKEKKLFQSQAGNHSLGEEEKITLSSFRIIRVIGSGAFGKVYLVNKKGTEEVYAMKVYDKMTLYEKNVILSTIGERNILKNMNSNFIVKLHYAFQAAQNLYLMMDFMVGGELYYHLKKAKKFDEERTKFYIAQVILAIECLHQNNIMYRDLKLENILLGQDGYIKLTDFGLSKEGIKDKDLTNTLCGTAEYMAPEQIMNEGHNKMADFWQIGVLTYEMLYGTTPFFNEFRPNKDEIFNDIIQGKYTFPQYFKDDAKNFIRGLLQKDVKKRLGYNGFHELKKHAFFADFDWDKLERQEIEPQFKPPLRSQVDLQNFNPRLVKNTMTDLGVKDNKPHLKEEIESQFRGFTFVKDDHLNTDVPQRNSSAERNN*

>CH445543

TTCAAAAATTTATTACTAGTTTAAAATATACATAGAAATACGATTTTAATCAAATTTCAAAATGGGAAATAGCCTTTGTATGGAACAAAAAGTTGAAAAGAATCAATAGAAGACAAAAAAATCAGGCTTGCAATCATCAAACAATGGTAATAATAAAAATTAAAGGAAAAATGAGAGGCTTAACTCGATTATAGATCTAAATGAATATAAGTTACCAGCAGACCCCAAGGAAAAAAAGCTTTTCTAGTCATAGGCAGGAAATCATTCACTTGGAGAAGAAGAAAAAATTACTCTCTCCTCATTCAGAATTATAAGAGTATTTAAACCTTTATTTTTGTTTCAATACGTTATTGATTTTGTTTATTTTTACTTACTTACTAAAGGTCATTGGATCAGGAGCTTTTGGTAAAGTGTACTTGGTAAACAAGAAGGGGACAGGTAATAAATTCAAAAAATATATAAAACAAATCTTTAAAAAACTCTTTATAAGTTAATCCATCAGCACTAAAAGATTAAAGTGCTAAGATGTTTTTGTTAACTCTATATTTGAAAGGGACTTTGATATTTGTTTATTAACCATTCTTTATTTATAAATATTTAATAGAAGAAGTGTATGCAATGAAAGTTTATGATAAAATGACATTATACGAAAAGAATGTAATTTTGAGCACAATTGGAGAAAGAAATATATTAAAGAACATGAATTCAAATTTCATCGTTAAGCTTCATTACGCATTTTAAGCTGCATAAAATCTCTATCTTATGATGGATTTTATGGTTGGAGGTAACAAAATAACTAAATTGTTTTTAGTTAAATATTTAGTTTTCAATAGCTATAATTTACAAGCTGTTAGAATTATATTATTATTTTTTTTTATATATGGTTTTTTTTTATTTTAAAATAATTATAGGAGAATTGTATTACCATTTGAAAAAGGCAAAAAAATTTGACGAAGAAAGAACCAAATTTTATATTGCTTAAGTTATTTTGGCCATCGAATGCTTGCACTAAAATAATATAATGTATAGAGATTTGAAATTAGAAAATATCCTTCTCGGATAAGACGGATATATTAAGCTCACTGATTTTGGTCTTTCAAAAGAAGGAATTAAAGGTAATAGAATCAATTTTTAGTTAAGGAGTTTTTAAAAATGGTCTTTGATACAAAATAGATTGTTTACAAGAAGAATTTATAAAAGAATAATCAATATTAAAAAAAGAGGTCTTAAATTAAAGTTAGTAGTTAAAAGTAATTTAGACGAATGATTTTGATTACCGTCAGATATTATTTGTTTATTATTAATAGTACGATTACAATTTAATTGATTCTAACAGGAAATACCAAAATATCGAATATTATTTAAAGCTGTAATCTCAGCATAATCTGAAGCATCATTTTTATGAGAAAATAATCTTTTTATAAGAAATTTAAAATAAATAAAAAAACTAACTGCCTAATTAATTTCAATAAAATTTGATTAAATTTATTCTATTTTATAATAAATATATTAAAATATACTATTATCCATCTTATAAAAAATAAATATTTTAATATCAAATAGACAAAGATTTAACAAATACATTATGTGGCACAGCAGAGTATATGGCACCAGAATAAATTATGAATGAAGGACACAATAAGATGGCTGATTTCTGGCAAATTGTAAATTTATTTAAAAAATTTAAAAATCTATAGAAATCAAAAAATA

TTHERM_00459230

model 23 ESTs Intron removal, leading to alternate start codon usage

MNDCYSDKEDNEEQNQIAQEEIFLVEMLATEGVNNNEIQKLKKNGILSLKSLVMNTKRDLVNIYGIPDNKADSYVKKASEILARSENSRLFSSEFVLGTTVLQRRSQIRRISTGSKALDDILNGGIESQSITEFYGEYRSGKTQIAHTACVLAQSQDHCQSPGKVLYIDTEGTFRPERICQIASHYGMEGEYALSNIIYGRAYNVDQQNTLLIKGAQLMVEENCFALLVVDSIMANFRCDFSGRGDLSERQQALGKFMSRLQRMAAEFNIAVIITNQVMADPSGAMTGGAIPQPKPIGGHILAHASTQRLFMKKKTDNIRKVKLVDSPYLQDKEVDIMVSDRGVGDVECDKKPSTG*

alternate 8 ESTs Retention of intron 2

MGEQSLKAQQNFMENIVQAKHKQLILHAFWLNLKIIAKVKQAQEILTGFKQIILLKYSHLNKKIGPGKVLYIDTEGTFRPERICQIASHYGMEGEYALSNIIYGRAYNVDQQNTLLIKGAQLMVEENCFALLVVDSIMANFRCDFSGRGDLSERQQALGKFMSRLQRMAAEFNIAVIITNQVMADPSGAMTGGAIPQPKPIGGHILAHASTQRLFMKKKTDNIRKVKLVDSPYLQDKEVDIMVSDRGVGDVECDKKPSTG*

>CH445543

TCAAAAAAAAATATTTTTATCATAAATATTCTATTTACATTTTAAGCAAATACCATCTTTAGGAAAATGAACGATTGTTACTCTGATAAAGAAGATAATGAAGAATAAAACTAAATTGCTCAAGAAGAGATCTTTTTAGTAGAAATGCTTGCAACTGAGGGAGTTAACAATAATGAAATACAAAAGCTTAAAAAGGTATGTAGTTGTTATATTAATTCACAATAAGACAATATCATTGATTATATAAAAATAAATATAAAGAATGGAATCCTTTCACTCAAATCTTTAGTTATGAACACAAAGAGGGATCTTGTTAATATCTATGGAATTCCTGATAATAAAGCTGATTCATATGTCAAAAAAGCTTCAGAAATATTAGCTCGATCTGAAAACAGCAGGTTATTCAGCAGTGAGTTTGTTCTCGGTACTACTGTACTATAAAGAAGGAGTTAAATAAGAAGAATATCAACAGGATCCAAGGCTTTAGATGATATATTGAATGGGGGAATAGAGTCTCAAAGCATAACAGAATTTTATGGAGAATATCGTTCAGGCAAAACATAAATAGCTCATACTGCATGCGTTCTGGCTCAATCTTAAGATCATTGCTAAAGTAAAACAAGCATAAGAAATTTTAACTGGTTTTAAATAAATTATTCTGCTTAAATATAGCCATTTAAATAAAAAAATAGGTCCAGGAAAAGTTTTATACATTGATACTGAAGGAACCTTCAGACCTGAAAGAATATGCTAAATAGCTTCGCACTACGGAATGGAGGGTGAATATGCCCTAAGTAATATAATTTATGGGAGAGCCTATAACGTAGATTAATAAAACACATTGCTTATTAAAGGAGCCCAATTAATGGTAGAGGAAAATTGTTTTGCTCTACTAGTTGTTGATTCAATAATGGCTAATTTTAGATGCGATTTTTCTGGAAGAGGAGATCTCTCTGAAAGACAATAAGCACTTGGCAAGTTTATGAGCAGATTATAAAGAATGGCTGCTGAATTTAATATCGCAGTGGTAAATAAATACATTCTTTATTTTATCAACACCTTTCTGCTTTTATACGATTCTTATTCTTAAAATTAACAAACAGATTATAACAAACTAAGTTATGGCTGATCCAAGTGGAGCCATGACTGGAGGCGCTATA

TTHERM_00522030

model 9 ESTs Intron retention

MNQNPLGSMKKGFEDINMYYNNQYFDFQQNQIGSILGQESNPLDQIKEKAEEQSNHGHGGQQISEGQPKQKPTNSKNTYIKKINNLVKTSYDNLQALDNKQDFNASQQDNSKMLRKTKSSYGLDLLNNQHQSNYLSQKNEFNSQNGLNHNQILAQQQQQQSQQMQGYSDPSMIQQQQQSLQNQNANPAKENQKLVRNRESARNSRKRKKIYLELLENKVTQLNDILQDSKRICCASEQLLNNLQTQIQYKNDQQTNKTILLNNLQNSLNSNASENDVGIIIEGLKRKFGSNNPERMMVLDYCFKQIAEQMLPVHMKYILYVASESKDIYSPDQDKDDELENQNKTQEFEFPKIIQSLKLSESQKKKAIKMQKKLSKEKEKLEQLVNNMYETKEKMKKELNSLDETMENLIKDFKPSQISKFLLSIERTQYNNHMKQAFQKFFEGDNDDSDDDSDNDLQSFIQNQDSCNTLMVDVNEAYDVYTDAYEFLQKKRHLSTDVNQTNLNAAAQAAFQNDSN*

alternate 2 ESTs Intron removal

MNQNPLGSMKKGFEDINMYYNNQYFDFQQNQIGSILGQESNPLDQIKEKAEEQSNHGHGGQQISEGQPKQKPTNSKNTYIKKINNLVKTSYDNLQALDNKQDFNASQQDNSKMLRKTKSSYGLDLLNNQHQTQQQQQQSQQMQGYSDPSMIQQQQQSLQNQNANPAKENQKLVRNRESARNSRKRKKIYLELLENKVTQLNDILQDSKRICCASEQLLNNLQTQIQYKNDQQTNKTILLNNLQNSLNSNASENDVGIIIEGLKRKFGSNNPERMMVLDYCFKQIAEQMLPVHMKYILYVASESKDIYSPDQDKDDELENQNKTQEFEFPKIIQSLKLSESQKKKAIKMQKKLSKEKEKLEQLVNNMYETKEKMKKELNSLDETMENLIKDFKPSQISKFLLSIERTQYNNHMKQAFQKFFEGDNDDSDDDSDNDLQSFIQNQDSCNTLMVDVNEAYDVYTDAYEFLQKKRHLSTDVNQTNLNAAAQAAFQNDSN*

>CH445667

AAAAAATATGAACTAAAACCCTTTAGGAAGTATGAAAAAAGGATTCGAAGACATTAATATGTATTATAATAACTAGTACTTTGATTTCTAATAAAATCAAATAGGCTCCATACTAGGCTAGGTATTTATCTAAAATAGTTTTGAATTAATATCATTAAATAACTTTACTTTGATTGTGTATTTAGAGCTAAAATAAATTAAGATTTTATATTTTTTAAATTTTAGGAATCAAATCCACTTGATCAAATAAAAGAAAAGGCTGAAGAGTAATCTAATCATGGCCATGGGTATTATATTAACTTTAATTATAGGAGAAATAAGAAAAAAAATATACTTTATTCAATTATTTAGAGGATAGCAAATTTCAGAAGGCCAGCCAAAATAAAAACCTACTAATTCTAAAAACACATACATCAAAAAGATAAATAATTTAGTAAAAACATCTTATGATAATCTTCAAGCACTAGATAATAAATAAGACTTCAACGCCTCTTAGTATTTAATTTTAAAATTTTAAGAAAGTGTGAGTAAAGTTAATGCTTTATAAAAAAATTAGATAAGACAACAGTAAGATGTTAAGAAAAACAAAGTCTAGTTATGGATTAGATTTACTTAATAATTAACACTAAAGTAATTATTTAAGTTAAAAAAATGAATTTAATTCATAAAATGGTTTAAACCATAATCAAATCTTAGCTTAATAGCAATAATAGCAGAGTTAGCAAATGTAAGGATACTCAGATCCTTCAATGATTTAATAGCAATAGCAATCTCTCTAAAACCAAAATGCTAATCCAGCTAAAGAAAACTAAAAGCTTGTCAGAAATAGAGAATCTGCCAGAAACTCAAGAAAGAGAAAGAAAATATATTTAGAATTGTTAGAAAACAAGGTAAAATAAATTTATATTGGAATTTTTATTTTTGTTTTAAATTGAACTATTTATATTTCTTTAGAACTAAAAAAATAACAAATAAATGAAATAAGTAAATAATATTTTAAGAGAAAATATTTGTATTAATAATTCATTAAAAATAAATAACTCTAAATTTCAAAAAGAAATTATTTTTACAGAAATTTATTTCTTAATTTAAATAATGCAAAATAATTGAACCAAGAAAAAAATAAATATTTCAAAATTTATAATGATTATAAATAAATAAATATTTATTAACTAAAGTATAGTTTTTTAATCATAATTTTAAAGTAGTTTTTATGATTTTTAAATTTAAATAGAATTAATTTAATTTAAATATTTATTTGGTGAGAATCAAATTATAAATATAAATTAAATTCATTTATAATAAGTATAATTAATTTTAATATTTAAGGTAACCTAATTGAATGATATTTTGTAAGACTCTAAAAGAATATGCTGTGCTAGTGAGTAGTTATTAAATAATTTATAAACATAAATTCAATATGTAAATTTTTTTGATCATTTTTGATTATTAATAAACTTTTAAAACAATTTTAATATAGAAAAATGACTAGTAAACAAACAAGACTATATTGTTAAATAATTTGCAAAATTCTTTAAACAGTAATGCTAGTGAAAATGATGTTGGAATTATTATAGAAGGTTTAAAGGTAAATAAAAATATTATTTTATAATTTAAAAATTTCAAAATGTTTATTTTCTTTAAAATTCATATTAGAGAAAGTTTGGTTCCAATAACCCTGAAAGAATGATGGTCCTTGATTATTGCTTTAAGCAAATAGCTGAATAAATGCTTCCCGTTCATATGAAATATATCCTTTACGTTGCCTCAGAGTCAAAAG

TTHERM_00476650

model 5 ESTs Intron retention

MSMEIEVDHSANIDVIDRYSPLGFRIPGFNDQSKIIEIVKQKFPENKGIEELRHPSNLTMDDLTRELKRENIIQHNQNIVYRDSYYYIDAIDHKTLSETEQAFDNAYDPLNNIPGDNETQNWKNNQPGIKLIQSKEQKTINGTQVTGAQYVLDGKLVQSGPAAAMAGATTNKNPNVIMQSHLTYQLTANPKSVIFEIKDPIILSTNLIEIFCKCQKSYEGELMIKCSNDNCKIKWFHPVCVGLGNINPRALEDLQFTCEDCKESERNIKKQSTNNGMDKASRKKATKN*

alternate 3 ESTs Intron removal

MSMEIEVDHSANIDVIDRYSPLGFRIPGFNDQSKIIEIVKQKFPENKGIEELRHPSNLTMDDLTRELKRENIIQHNQNIVYRDSYYYIDAIDHKTLSETEQAFDNAYDPLNNIPGDNETQNWKNNQPGIKLIQSKEQKTINGTQVTGAQYVLDGKLVQSGPAAAMAGATTNKNPNDPIILSTNLIEIFCKCQKSYEGELMIKCSNDNCKIKWFHPVCVGLGNINPRALEDLQFTCEDCKESERNIKKQSTNNGMDKASRKKATKN*

>CH445626

GCGAAACTTAGAACTGGAAAAATAACTAGCCAGGCATTAAGCTAATCCAATCAAAAGAATAAAAGACAATAAATGGCACATAAGTTACTGGAGCCCAATACGTATTAGATGGAAAACTGGTTTAAAGTGGCCCAGCAGCAGCAATGGCAGGCGCAACTACGAATAAAAATCCCAATGTAATTATGCAATCACATTTAACTTATTAATTGACAGCTAATCCAAAGAGTGTAATTTTTGAAATTAAGGATCCAATCATTTTGAGTACAAATCTCATTGAAATTTTCTGCAAGTGCTAAAAATCATACGAAGGAGAACTGATGATAAAGTGTTCAAACGATAACTGCAAGATAAAGTGGTTCCACCCTGTTTGTGTAGGTCTTGGAAATATAAATCCAAGAGCTCTTGAAGACTTATAATTTACTTGTGAGGATTGTGTAAGATTATTGAAATAATGCTTTAATTTTATATCTTTATTTTTTAAATAATATTATAGAAAGAATCCGAAAGAAATATCAAAAAACAATCTACGAACAATGGTATGGATAAAGCTTCACGTAAAAAGGCAACCAAAAATTGATAATCAAGTCTAATATGAATCAAGCAAATCTTATATTGATTAAAATCTA

TTHERM_00418290

model 3 ESTs

MEIATEVRDVYTFSPGPCSLPLGVQRSCHNSLWNFEDLGYGSLEIPGNSYESKILVKKCKDNLRTLFELPDNYSVMLMEGGAHLLNSGIPLNMIPEGGSANYLVTGFWGARTHKESLKFGNIKLVHEIVPQMNYIPDEKDWQIDTKGSYFHFTDNETLSGLEFKQVPYAQGQNIVADMTSSLGTKKLETNKYAVIYAAAQKNLGIAGNTVAFVRNDLIGKPQKMTPSYMDWRNMVDENFDYNMGIYSIYATNTYVEYLNQAPGKLDYWENLANQKAKLIWDVIDGSRGFFKPLCTKRDQRSRLNITFYCANDEKIDNLFIEEAAKIGLIELKGHPATKGVRASIYNGTQLEGVKKLRDFMLDFQEKNEVRIVNTLSRL*

alternate 2 ESTs Intron removal

MEIATEVRDVYTFSPGPCSLPLGVQRSCHNSLWNFEDLGYGSLEIPGNSYESKILVKKCKDNLRTLFELPDNYSVMLMEGGAHLLNSGIPLNMIPEGGSANYLVTGFWGARTHKESLKFGNIKLVHEIVPQMNYIPDEKDWQIDTKGSYFHFTDNETLSGLEFKQVPYAQGQNIVADMTSSLGTKKLETNKYAVIYAAAQKNLGIAGNTVAFVRNDLIGKPQKMTPSYMDWRNMVDENFDYNMGIYSIYATNTYVEYLNQAPGKLDYWENLANQKAKLIWDVIDGSRGFFKPLCTKRDQRSRLNITFYCANDEKIDNLFIEEAAKIGLIELKGHPATKGVRASIYNGTQLEGVKKLRDFMLDFQEKNEVKQ*

>CH445785

CTCAACTAAGCTCCAGGCAAACTTGATTATTGGGAAAACCTTGCAAATTAAAAGGCTAAATTGATTTGGGTAAAATAAAAATTATTAAATTAATATTGAATACTAATTAATTTATAATAGGATGTTATTGATGGTTCAAGAGGTTTCTTCAAGCCACTTTGTACCAAGAGAGACCAACGTTCAAGATTGAATATTACTTTTTACTGTGCAAATGGTACTATTTTTTTTTAATTTTATGAAAAACAAATTGTTAATTTTATTTATAGATGAAAAAATAGATAATTTATTCATTGAAGAAGCTGCAAAGATTGGTTTAATTGAATTGAAGGGACATCCTGCTACAAGTATTAATTAATTTTCTTGATATATTTTTGAATTTACTAATTTTATTTTTATTAACTTATTAAAGAGGGTGTAAGAGCTAGCATTTATAATGGTACTCAACTCGAGGGCGTTAAAAAGCTTAGAGATTTCATGCTCGATTTCCAAGAAAAGAATGAGGTGCGAATTGTTAATACTTTATCAAGACTCTGATTTTTTTTAAATAGTTTTTTAATGAATTTATAAATTAAACTAAAATAGATATTAATAAAAAAATAACAACAAAAAATTAATTAATAATAACCATTTTCAAACAAAACAAAAGAATATGTAGATATTATTCAACTAAAGGACTCAAAAAGCAATAAATAGCTAATAATCTATCTATCTATCTGTCTAAATAACTAAATAACTAGCTTTACTACCACTAATAATGACATATAAATTTTACAATTATTAGAAGAATATTTTTTATACAATACAATACTACTTATTTTTTCTTATATATTATATTAAAATAAAATTTATATAAAATAAATAAATCCTTAAAGGTTAAATAATGAAGTTGAAACTTTGCATATTCTCATATCTTTCACTTGTACCTCCTTATTCATACTATTGATTAACTA

TTHERM_00637820

model 2 ESTs

MQAKKPLGFWQTKTDLLKKYKDEKRKKLDRFKKDKKSLNSDMDTSNTSSGGNNISKTLLSGKATSDSVTFEMSELPPIWVEIHHQTENLLKEIVDIKKDIIKESAIRIRRQFNDNGELDNKINNLVQVAMKKIKEAEANILKIDKLAEKTQETDQEKRIRQNVKLSLASQIQELTVDFRRQQKGLYDQLKQYNNVGQTGFMHQIDYTQQDQMMQDQDMYEQIARDRDAEINKIVDMINELSSIYQQLGHLVLETGTLIDRIDFNITQAKENTQKANVHLKKTVQYQESPTAKRCVQILIILIIIFAFILTLKYK*

alternate 2 ESTs Alternate 3’ splice junction

MQAKKPLGFWQTKTDLLKKYKDEKRKKLDRFKKDKKSLNSDMDTSNTSSGGRKATSDSVTFEMSELPPIWVEIHHQTENLLKEIVDIKKDIIKESAIRIRRQFNDNGELDNKINNLVQVAMKKIKEAEANILKIDKLAEKTQETDQEKRIRQNVKLSLASQIQELTVDFRRQQKGLYDQLKQYNNVGQTGFMHQIDYTQQDQMMQDQDMYEQIARDRDAEINKIVDMINELSSIYQQLGHLVLETGTLIDRIDFNITQAKENTQKANVHLKKTVQYQESPTAKRCVQILIILIIIFAFILTLKYK*

>CH670354

AGATTTAAGAAAGATAAAAAATCTCTAAACTCTGATATGGATACATCGAACACAAGTAGTGGAGGAAGTAACCAATAATCTTTATTATATAATTGGATTTATTAAAAATTATATAGATAATATATCTAAGACTTTATTGTCAGGAAAAGCAACATCCGATTCGGTCACATTCGAAATGTCAGAACTCCCTCCTATTTGGGTTGAAATACACCACTAGACAGAAAATCTTTTAAAAGAAATAGTTGATATAAGTTAATATTTTTTCTTTTTGTTTTCATTTAATTTATTGATTGATTGTGGTTTTATTCACAATACTTGTTTGGCGTTAAAGTAAAAAAAACCTTTGATTTCTAAAATAAAATAGAAAAAGATATAATAAAAGAGAGTGCAATCAGGATCAGAAGATAGTTCAACGATAATGGAGAATTAGATAATAAAATAAATAATTTAGTTTAAGTCGCAATGAAAGTAAATTATAAATCAAATTTATTTATTTTATGCATATTAGTAGAATAAATTCATTAATATTAAATTATATAAATTATATAATAATGAGTGAATTTATTTTATTATTTTTAGAAAATCAAAGAAGCCGAAGCCAACATCTTAAAAATAGATAAGTTAGCCGAAAAAACATAAGAAACTGACCAAGAAAAAAGAAGTAAAATTAATAGATATTTTGTCATGATTTTAGATAGAAAATACATTATATTCAATAAAAAGTTAGATAAAATGTAAAGCTTTCATTAGCATCTTAAATTTAAGAGCTTACTGTAGACTTTAGGAGATAATAAAAAGGATTATATGATCAGCTTAAGTAATATAATAATGTTGGTTAAACAGGTTTTATGCATTAAATCGACTACACCTAACAAGATTAGATGATGCAAGACTAGGATATGTACGAACAAATAGCAAGAGATAGGTAAAATAAATTTTAAATTTCGTTGAGATTAATAAATCTTAAAAATTAGAGATGCTGAAATAAATAAAATTGTTGATATGATTAACGAGCTGTCAAGTATTTACCAGCAATTAGGGCATCTAGTACTAGAAACTGGTACTTTAATTGATAGAATTGACTTTAATATAACTTAAGCTAAAGAAAATACTTAAAAAG
